# Diagrammatic physical robot models

**DOI:** 10.1007/s10270-025-01270-9

**Published:** 2025-03-12

**Authors:** Alvaro Miyazawa, Sharar Ahmadi, Ana Cavalcanti, James Baxter, Mark Post, Pedro Ribeiro, Jon Timmis, Thomas Wright

**Affiliations:** 1 Department of Computer Science, University of York, York, UK; 2 Department of Meteorology, University of Reading, Reading, UK; 3 School of Physics, Engineering, and Technology, University of York, York, UK; 4 Department of Computer Science, Aberystwyth University, Aberystwyth, UK; 5 Department of Electrical and Computer Engineering, Aarhus University, Aarhus, Denmark

**Keywords:** Simulation, Verification, SDF, Hybrid models, Diagrammatic models

## Abstract

Simulation is a favoured technique in robotics. It is, however, costly, in terms of development time, and its usability is limited by the lack of standardisation and portability of simulators. We present RoboSim, a diagrammatic tool-independent domain-specific language to model robotic platforms and their controllers. It can be regarded as a profile of UML/SysML enriched with time primitives, differential equations, and a mathematical semantics. Our previous work on RoboSim described a notation to specify control software. In this paper, we present a novel notation to describe physical models: block diagrams that can be linked to the platform-independent software model to characterise how services required by the software are realised by actuators and sensors. Behaviours are specified by differential equations, and simulations and mathematical models of the whole system can be generated automatically. Our main contributions are a modular and extensible diagrammatic notation that supports the explicit specification of physical behaviours; a set of validation rules that identify well-formed models; a model-to-model transformation from RoboSim to an input format accepted by several simulators; and a formal semantics for mathematical reasoning.

## Introduction

The importance of rigorous approaches to software engineering in robotics is recognised, but it is still a new and developing research area [15]. There are a number of challenges: (1) the development of software is often tackled at the programming, rather than modelling level; (2) the APIs of robots and simulators differ significantly and require manual adaptations; (3) simulations often rely on black-box simulators where assumptions and physical equations are not readily available; and (4) verification is by trial and error [10]. All this drives costs up, with the need for expensive tests and to deal with low-level code. Moreover, in the end, the only guarantee is that undesirable behaviours have not been found, not that they are not possible. Here, we propose a new notation to support modelling and verification of a robotic system based on simulation and proof, advancing previous work by tackling models and properties that involve the physical hybrid model of the robotic platform.

Several reactive simulators are available [42, 50, 61]. They typically, however, use dedicated APIs, with different libraries to support simulation of platforms and environments, sometimes written using proprietary languages. Lack of portability has an impact on the cost of simulations and availability of simulation facilities. In addition, although extremely useful to explore the design of systems, simulations are tests. In our approach, costs are lowered by automatic generation of (simulation) code, and confidence is increased by complementary proofs.

Our work is based on RoboSim, a diagrammatic notation for tool-independent modelling of robotics simulations. With the work presented here, modelling can cater for both control software and hardware designs. This provides support for simulation and proof of properties [52] that may not be true, or even expressible, when the software is considered in isolation, but are valid when the physical platform is in the loop.

Previously, we have presented the RoboSim notation for modelling software [19]; it can be regarded as a profile of UML [58] component diagrams and state machines. Over and above what can be specified in UML, however, RoboSim supports the definition of timed properties backed by mathematical models automatically generated for verification. With a mathematical model, we can, for instance, prove properties observed in a simulation or compare a RoboSim (simulation) model with a design model, to ensure consistency.

The work presented in this paper enriches RoboSim with a profile of block diagrams of the systems modelling notation SysML [57] to describe physical models of robotic platforms. With this facility, we can define an integrated model of a controller software and the physical platform that it controls. RoboSim block diagrams adopt concepts of XML-based notations used by robotics simulators, notably, SDF (Scene Description Format).^1^ So, in terms of ease of use, we benefit from the popularity of both SysML and SDF.

SDF can be used to describe scenarios, including, but not restricted to, robotic platforms. It is accepted by popular simulators, such as, Gazebo [42] and CoppeliaSim [61]. It is a superset of URDF (Unified Robot Description Format), adopted by the widely used ROS (Robotic Operating System),^2^ and accepted by, for example, Simulink.^3^ Popular physics engines^4^^,^^5^ work with SDF documents. SDF embeds domain knowledge in the form of XML tags to describe elements of robotic platforms and environments. The use of SDF avoids or minimises the need to program a simulation of a customised platform. Alternative formats, such as COLLADA,^6^ exist but are more general and focus on 3D models instead of the specificities of robotic applications.

SDF, however, is a textual notation without clear semantics. The behaviour of an SDF document is defined by the particular physics engine adopted in a simulation. In [65], a number of challenges in the adoption of URDF are identified, and while SDF addresses some of these challenges (for instance, modelling parallel linkages), others such as XML syntax and ensuring accuracy of frames remain. Moreover, there is no support for validation of SDF documents, beyond graphical rendering in a simulator, which may not be possible for an invalid document.

In contrast, RoboSim is a diagrammatic notation with a specialised editor, which also checks the validity of models. Moreover, RoboSim supports model decomposition for readability and reuse, and the definition of new kinds of sensors, actuators, and joints. Importantly, RoboSim supports behavioural modelling, building on a uniform and simple approach to specify frames of reference and poses. Finally, RoboSim’s process-algebraic semantics allows verification of properties related to both the control software and the physical robot. Our focus is on the rigid-body dynamics of the robots [46, 64] (and their interaction with the software) and not on aspects such as soft bodies, stress, and strain. It is worth noting, however, that while we use formulations of robot behaviours from sources such as [46] in our examples and libraries, RoboSim is agnostic to the formulation of these behaviours. The only two limitations are the focus on rigid bodies and the use of roll-pitch-yaw poses. Rigid bodies already pose challenges (addressed here), and roll-pitch-yaw poses simplify the translation to widely used notations such as SDF. Both restrictions can be relaxed and are the subject of planned future developments.

The RoboSim block diagram notation presented here includes a significant number of extra facilities for modelling and reuse, and yet can be used to derive an SDF document. In this paper, we present model transformation rules that we have implemented to automate the generation of SDF documents for CoppeliaSim [61]. The SDF documents generated can be used directly, or tailored to specify how aspects of a particular scenario (wind, light, and so on) affect the system.

In addition, RoboSim models record (a system of) differential equations that characterise the behaviour of sensors, actuators, and joints in terms of their inputs and outputs. The equations provide a mathematical description of the physical properties of a platform. When SDF is used with a simulator, the sensors, actuators, and joints are simulated using a physics engine that typically provides code for efficient approximate accounts of this behaviour. In RoboSim, the equations can provide mathematical accounts of (continuous) behaviour useful for mathematical proof.

We have defined and implemented model transformation rules that define a process-algebraic semantics for our RoboSim block diagrams. Due to the continuous nature of physical systems, we need to account for both the discrete behaviour of the software and the continuous behaviour of the robotic platform. So, we use a data-rich hybrid version of the process algebra CSP [62], called *CyPhyCircus* [53]. The semantics of RoboSim software models [19] is already described in CSP [62]. So, as we illustrate here, that semantics and the *CyPhyCircus* semantics we present integrate well to provide a unified model of the control software and of the robotic platform.

On the other hand, while RoboSim block diagrams are described here in the context of RoboSim control software models, the notation is general. RoboSim block diagrams can be combined with platform-independent software models written in other languages. For instance, in [17], RoboChart [19], the notation for software modelling on which RoboSim is based, is combined with SysML to support simulation and co-simulation, and in [54], RoboChart and Simulink [47] are combined for co-verification of a high-voltage electrostatic control system.

In RoboSim, the connection between a block diagram and a control software model is defined by another diagram: a platform mapping that specifies the interaction between them. With this separation, the generation of simulations and of the formal semantics can be tackled in a self-contained manner. For verification purposes, the semantics of RoboSim p-models can be used in isolation or composed with other models as long as a suitable formal connection is established. So, verification relies on the software model only when the property of interest relates to and depends on the behaviour of both the software and the platform.

RoboSim is supported by a tool called RoboTool.^7^ We have extended it to support RoboSim block diagrams. With RoboTool, we can validate the models to ensure that they are well-typed and well-formed. We can also generate automatically SDF documents for simulation and mathematical models for mechanised proof of properties. All examples in this paper have been developed using RoboTool, as have the physical model for the complete marXbot [9]^8^ (a robot with several modules), a dressing robot, a firefighting drone, and a solar panel vacuum cleaner [2].

To summarise, our novel contributions here are: (1) a modular and extensible diagrammatic notation for the physical modelling of robotic platforms that supports the explicit specification of physical behaviours while improving readability; (2) conditions that characterise well-formed models; (3) model transformation rules for automatic generation of SDF documents from diagrams for simulation; (4) model transformation rules for automatic generation of *CyPhyCircus* diagram models for proof in the context of a control software; and (5) a tool for editing and validating models, and automatic generation of SDF documents and mathematical *CyPhyCircus* models. To illustrate our approach to proof, we use an integration of two model checkers: FDR (for discrete time software models) [33] and Flow* (for hybrid platform models) [23].

Our vision for the development of robotic applications follows an iterative pattern where initially software and physical models are created and properties are specified, and at each iteration, the properties are verified, simulations and tests are automatically generated and executed, code is generated and deployed, and real-world tests are run. Any problems identified in verification, simulation, or deployment are addressed directly in the models, which are updated and re-evaluated in the next iteration of the development process. This approach complements current practice by providing further structure to the development process and improving automation and reuse.

Next, we discuss related work. Section 3 gives an overview of control software modelling in RoboSim. Section 4 describes our novel approach to physical modelling; we define the metamodel of the block diagrams and their well-formedness conditions, and provide examples. Section 5 describes the approach to generate SDF documents, and Sect. 6 shows the approach to generate mathematical models for proofs. RoboTool and the implementation of our notation are discussed in Sect. 7, and several case studies are described in Sect. 8. We conclude and indicate future work in Sect. 9.

## Related work

There are several general-purpose languages that can be used in robotics. Some of them are diagrammatic, notably UML and its variants (SysML, UML MARTE, AADL, and so on). For most, there is some support for verification and code generation, but not for physical modelling, which is our focus here.

We can find several domain-specific languages for robotics in the literature [56]. They target control software [15]. Automatic generation of code is a main motivation for their design, and in several cases, the generated code can be used for simulation. There is, however, no coverage of code to simulate the robotic platform as we do here. A few languages support verification [29, 32, 35] of software properties. With RoboSim, we can also reason about properties that are related to, or depend on, the physical platform.

Closest to our work is that in [3], in which a UML profile caters for modelling scenarios where robots collaborate with humans. Scenarios are captured by class diagrams and can specify physical components of the robot. There is a notion of mobile device with attributes relevant for reasoning about the safety of collaborative applications. A mobile device has a type (anthropomorphic, SCARA, cartesian, or dual arm), DoF (Degree of Freedom), joints (prismatic, rotational, or spheric), maximum weight it can carry, and maximum reach. Skills (moving, hooking, and so on) can be recorded. Specific types of end-effectors can be identified (grippers, screwdrivers, and others), with relationships (such as ‘attached to’) capturing some kinematic restrictions. Control is described via activity diagrams, picturing the actions of the human and the robot. Mathematical models for verification automatically generated use a temporal logic with a notion of discrete time. We share the vision to automate artefact generation to deal cost-effectively with changes. Their goal, however, is different: risk analysis and generation of deployment code with a focus on human interaction. Their models go further than ours, covering the behaviour of operators, but do not cater for concepts such as controllers, sensors, and actuators, and their hybrid behaviour.

A version of UML for mechatronics, called mUML, is presented in [34]; its focus is modelling and verification for systems with reconfigurable software. Like RoboSim, it uses a specific component model and has support for model checking. An mUML model follows a hierarchy, which, at the lowest level, captures alternative control strategies defined by differential equations. Physical modelling, however, is not considered in [34]. Joint use of RoboSim block diagrams, as presented here, and mUML software models can enable the use of their tool for code generation. In general, RoboSim block diagrams can be used to complement software models written in other languages for robotics in the literature, especially those few that define a mathematical model for the software.

A popular line of work is the use of mathematical approaches to develop planners [12, 43, 66]. An extensive survey [28, 45] indicates that model checking is the most popular approach for specification and verification in robotics. RoboSim brings together the convenience of diagrammatic modelling and the power of mathematics. Since mathematical models are generated automatically, specialised modelling expertise is not required. Yet, the mathematical models generated open a wide variety of possible avenues for verification.

Automated proof (via model checking) and physical modelling has been pursued using JavaPathFinder [63]. Physical modelling is via (linear time-invariant ordinary) differential equations and covers the robotic platform and the environment. The control software is written in Java. The equations capture inputs to sensors and outputs of actuators, but abstracts the physical structure of the robot. Model checking uses a separate simulation of the dynamical systems in conjunction with the Java code. In contrast, we automatically generate simulations and mathematical models.

The RoboSim mathematical model of a robotic platform is generated based on the behaviour of its sensors, actuators, and joints, and on properties of its components. An early work [59] on modular robotics uses data stored in modules to obtain the gravity vector. Approaches based on Lie groups [22, 48, 60] are suitable for modular structures with particular geometries. An approach based on the standard Denavit–Hartenberg (D-H) convention [7, 8, 27] is used in [40] specifically for revolute joints, and in [64] for consecutive axes.

Property-driven approaches [11] use models to guide the construction of a simulation. Similar to ours, their engineering approach benefits from precise mathematical models for proof. Their model is based on automata, and gives a monolithic account of the system as a whole. Simulations have to be developed by hand. Use of RoboSim models to generate mathematical models and simulations automatically can reduce costs by avoiding the manual development of simulations.

In Capella,^9^ physical modelling refers to allocation of software components to computational units. This is important, but complementary to our work, we concentrate here on the physical behaviour of mobile robots. An example where a CAD tool is used for physical modelling in conjunction with CoppeliaSim is in [24].

Xacro^10^ is an XML macro-language used as a front-end to simplify URDF descriptions. Use of Xacro increases modularity, reduces redundancy, and permits parametrisation. Using Xacro, we can obtain automatically generated URDF descriptions. The design of Xacro shares many of our goals. RoboSim, however, goes further in adopting domain-specific concepts (links, joints, parts, and so on) as building blocks, rather than XML macros. RoboSim also supports the definition of equations to capture behaviour (of sensors, actuators, and joints) and has a formal semantics. RoboSim is not a front-end for SDF or URDF.

Use of SDF for physical modelling is described in [68]. Automatic generation of SDF documents is reported in [41]. The source is an object-oriented language for building modelling called BIM. A technique for construction planning and scheduling based on BIM is integrated with ROS for generation of task plans for robots. In this approach, the spatiotemporal information of a building and its construction plans are used to define an SDF model for the building. The model for the robot is defined (by hand) using URDF.

In summary, RoboSim is a tool-independent domain-specific diagrammatic simulation language for robotics with distinctive features. It has support for generation of SDF documents and mathematical models. The work presented here enriches RoboSim to cover physical modelling of robots. With this, for verification, we can generate simulation code for the software and the robot, and prove properties that depend on both. So, the RoboSim notation now supports the definition of three aspects of simulation models: software, robotic platform, and their connections.

**Fig. 1:**
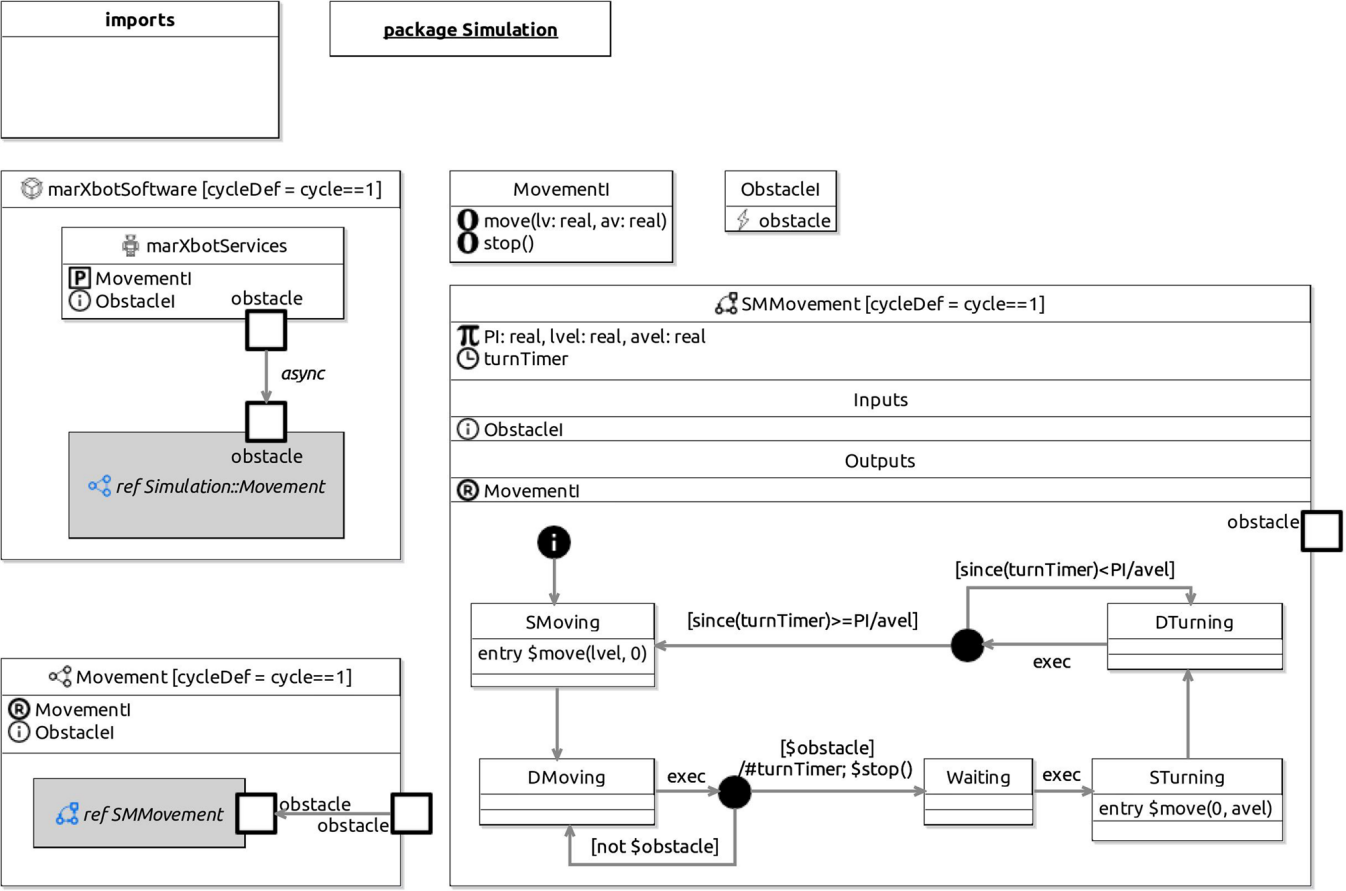
RoboSim: obstacle detection control software—module marXbotSoftware defines the control software as a whole, in terms of a robotic platform marXbotServices and a controller block Movement, whose behaviour is defined by a state machine SMMovement. MovementI and ObstacleI are interfaces used in the definition of the various components

## RoboSim control software

RoboSim is part of the RoboStar [13] family of notations. It enforces a cyclic behavioural pattern, where the system evolves in steps in which, first, inputs are read, and then outputs are calculated and provided, all infinitely fast, before time advances to the next cycle. The subset of RoboSim concerned with models of control software is called d-models[19], and the semantics of RoboSim d-models supports the comparison between RoboSim models and a reactive design model. (In RoboStar, reactive control software models are written using RoboChart.)

We give in this section a brief overview of how control *software* is modelled and verified in RoboSim. For that, we use the toy example in Fig. 1: a model for a robot that detects and moves away from obstacles. Figure 3 shows on the left the marXbot [9], a robot that can be used for this simple application. The marXbot is a modular mobile robot for swarm applications that uses differential-drive treels to deal with rough terrain, and includes a powerful battery and high-quality cameras. Complete physical models of the marXbot and of other commercial robots, described using the RoboSim diagrammatic notation presented in this paper, are available.^11^

**Fig. 2:**
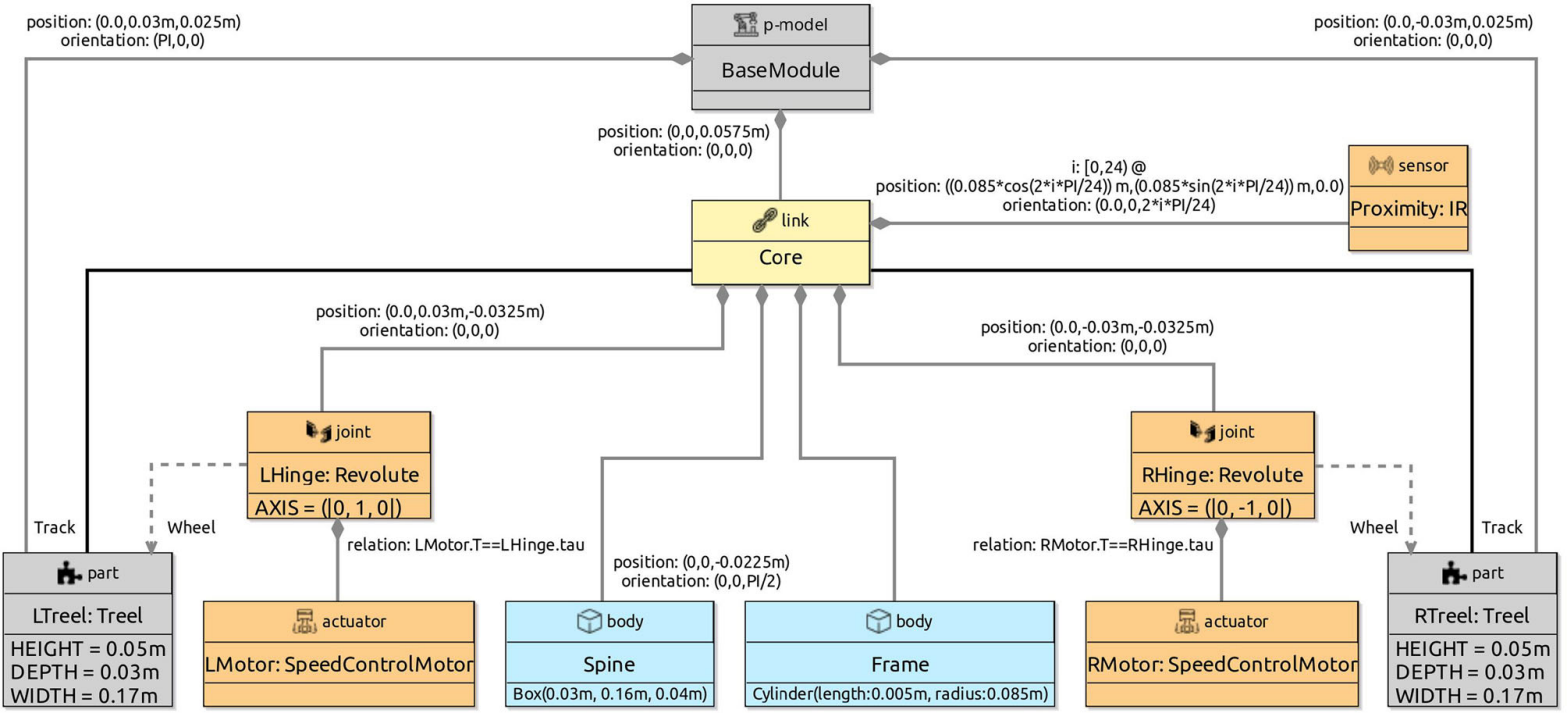
p-model of the base module of the marXbot [9]

In the next sections, we use as a running example the physical model in Fig. 2 just for the marXbot base, which is enough for the application defined in Fig. 1. Since this is a RoboSim d-model, it captures a software simulation model and the requirements that it imposes on the robotic platform, not a specific platform. A full account of RoboSim software models is in [18, 19]. Table 1, explained later, describes how the requirements on the robotic platform defined in Fig. 1 can be satisfied by the model in Fig. 2.

Control software is described in RoboSim by a module. In Fig. 1, the module for the simulation of our simple robot is given by the block named marXbotSoftware. The declaration (at the top) of the module includes a cycleDef clause that defines the length of the simulation cycle; in our example, it is 1 time unit. The specific value of a time unit is defined as part of the automated process to generate simulation code.

A module includes a component, namely the robotic platform that specifies the services required by the software. These requirements are defined by variables, events, and operations that are realised by sensors and actuators. The variables, events, and operations define an abstraction of the sensors and actuators. In our example, we call this abstraction marXbotServices.

In the definition of marXbotServices, we declare a provided (P) interface MovementI and a defined interface (i) ObstacleI. MovementI declares two operations move(lv:real,av:real) and stop() to capture services provided by embedded software in the marXbot using its motors. With a call to move(lv:real,av:real), we request that the linear and angular velocities of the robot are set to lv and av. With stop(), we request the robot to stop. These operations are provided by marXbotServices for use by the control software. In the module, these operations are not further defined.

The event obstacle defined in ObstacleI is an abstraction for an embedded operation that uses one the marXbot infrared sensors to signal the presence of an obstacle. Other forms of sensor may be used in other platforms. The event is used by the control software to make decisions, but is not further defined by the module.

The realisation of the variables, events, and operations declared by a robotic platform block are defined as part of the physical model in terms of its sensors and actuators, using the new notation for platform mappings presented in this paper. In the definition of the modules (that is, of software controllers), the exact physical robotic platform used, including the exact sensors and actuators that it includes, is not relevant. In a module, the services provided and defined by the robotic platform, as characterised by the variables, events, and operations, define a data model. For this reason, we call a module a d-model, standing for data model, to distinguish it from the physical models, called p-models, we describe here.

Besides a robotic platform, a module includes controllers that define the software behaviour using state machines. In our example, marXbotSoftware includes just one controller, called Movement. The connection (represented by an arrow) between the platform marXbotServices and Movement indicates that Movement uses obstacle as an asynchronous input. Movement can also call the provided operation move(lv:real,av:real).

Figure 1 presents the definition of Movement and of the machine SMMovement that defines its behaviour. In general, RoboSim permits the definition of parallel controllers whose behaviour can itself be defined by several threads of execution specified by parallel machines. For our purposes here, however, the details of how a d-model can be defined are not important. In our example, we briefly note only the uses of move(lv,av), stop(), and obstacle in SMMovement. In the initial state SMoving, the operation call move(lvel,0) is used to get the robot to move in a straight line with speed lvel. Afterwards, SMMovement changes to the state DMoving. Now, after each cycle of simulation, marked by a special machine event exec, a decision is made based on whether the event obstacle has happened or not. When it does, in the action of a transition to a state Waiting, the operation stop() is called (after a clock turnTimer is reset). Next, in the following cycle, marked by the exec event in the transition to another state STurning, the call move(0,avel) requests that the robot turns. Afterwards, in each cycle, we determine whether enough time, namely PI/avel time units, has passed, by checking the value of the clock since it was last reset, given by since(turnTimer). When enough time has passed, a transition leads back to the state SMoving, where the robot is again asked to move in a straight line (move(lvel,0)).

RoboTool automatically generates the semantics of a RoboSim d-model (as well as of a p-model as described in Sect. 6). Using that mathematical model, we can carry out verifications (in the way explained in [52]). For example, using the d-model we have proved the following property.

*Once an* obstacle * is detected, the operation* stop * is called immediately.*

This is true in our software design: In the same cycle of the simulation in which the event obstacle is raised, the operation call stop() takes place. Of course, the robot does not actually stop immediately, and several factors play a role in the delay. With the d-model, we cannot consider any of these factors. With a p-model, we can consider such issues in two ways. First, we can automatically generate a simulation that can be used to test the system in several scenarios. In addition, we can generate a richer mathematical model that captures, for example, the power and inertia of the motors, and other attributes of the platform. This model can be used to prove properties taking into account physical aspects of the system.

**Table 1:** RoboSim: connection between the software (marXbotSoftware in Fig. 1) and physical (BaseModule in Fig. 2) models, and the variables characterising the visible behaviour in the environment: torque of the two motors (LMotor.tau and RMotor.tau) and distance of obstacles (Proximity[1].distance)

marXbotServices from d-model	BaseModule—p-model	Environment
move(lv:real,av:real)	LMotor.das and RMotor.das	LMotor.tau and RMotor.tau
stop()	LMotor.das and RMotor.das	LMotor.tau and RMotor.tau
obstacle	Proximity[1].voltage	Proximity[1].distance

To summarise, Table 1 lists the events and operations of marXbotSoftware. A p-model is defined in Sect. 4 (Fig. 2) to illustrate the notation introduced in this paper. That model, called BaseModule, describes a robot with two motors, named LMotor and RMotor, and twenty-four infrared sensors Proximity. Table 1 indicates that the inputs das of the motors (defining the desired angular speed for the wheels) and the output voltage of one of the sensors are used to specify the operations and event of marXbotSoftware.

Together, the d-model (module) marXbotSoftware in Fig. 1 and the p-model BaseModule in Fig. 2 give an overview of the robotic system. The visible behaviour specified by the overall model affects and is affected by the environment. It is characterised by the outputs tau of the motors, recording the torque it induces, and the input to the sensor that records the distance to an obstacle. The definition of how the various quantities, events, and operations in Table 1 are related is also recorded in the p-model (see Fig. 8).

**Fig. 3:**
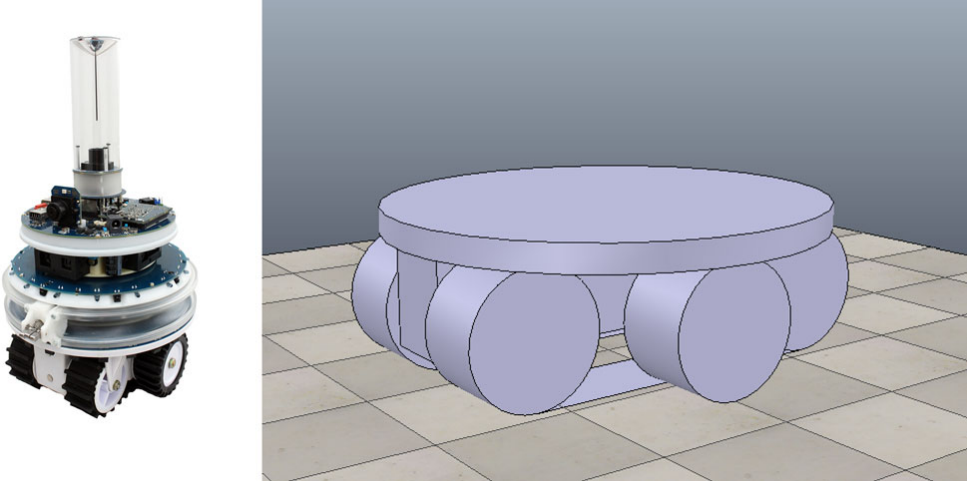
Real marXbot [9] and the rendering of BaseModule—using SDF in CoppeliaSim

With the p-model, we can automatically generate a simulation using the platform rendered in Fig. 3, and prove the following property, in terms of quantities related to environment factors, not software components.

*Once the* distance * between the platform and an obstacle is less than a value* d *, after* t * time units, the values of* tau * output by the motors are 0.*

In the next section, we describe our novel notation to define and use RoboSim p-models.

## RoboSim physical models

Here, we present the RoboSim block diagrams used to specify p-models, first via examples (Sect. 4.1), and then their metamodel (Sect. 4.2), and the well-formedness rules that characterise valid p-models (Sect. 4.3).

### Overview

In this section, we provide an overview of RoboSim block diagrams focusing on the five main aspects of the notation: the basic syntactic elements (Sect. 4.1.1); the different types of blocks (Sect. 4.1.2); the mechanisms for structuring models and extending the language (Sect. 4.1.3); the connection between physical and software models (Sect. 4.1.4); and the facilities for annotating models (Sect. 4.1.5).

A RoboSim block diagram can be used to define a physical model for a robotic platform (called a p-model as mentioned above) to capture its physical elements, such as its links, that is, its rigid bodies, joints, sensors, and actuators, and their behaviours. A robotic platform in a module can be associated with a physical description via a connection between the module and a block diagram. A property of that connection is a platform mapping, a separate block defining how the variables, events, and operations of the robotic platform in the module are realised by the sensors and actuators of the p-model defined by the block diagram. A platform mapping contains a mapping for each element (variable, event, and operation) of the robotic platform in the d-model.

In our example, as said, the operations of the robotic platform marXbotServices (see Fig. 1) are realised by actuators, and its event obstacle is realised by a sensor (see Table 1). Figure 2 presents a RoboSim block diagram for a p-model called BaseModule that we use to describe a possible physical realisation of marXbotServices. The whole marXbot is shown in Fig. 3 on the left, and its p-model, which uses BaseModule, is available.^12^ On the right, Fig. 3 shows a rendering for our p-model (just for the base) in Fig. 2.

#### Diagram elements: blocks and connections

***Blocks***

A block can represent a p-model as a whole, a link, a joint, a sensor, an actuator, a body, or a part, itself defined by a separate block diagram. Different icons identify the various kinds of blocks in a RoboSim diagram:  for a p-model,  for a link,  for a joint,  for a sensor,  for an actuator,  for a body, and  for a part. These blocks are described later in this section.

***Connections***

Table 2 lists the forms of connections available in block diagrams and their representation. The containment relationship is represented by a solid line with a diamond on the side of the block that represents the containing element. In Fig. 2, BaseModule contains a link Core, and two parts named LTreel and RTreel defined later by another p-model called Treel (see Fig. 7).

**Table 2:** Connections of the RoboSim block diagrams

Connection type	Description
Containment	Diamond solid line
Flexible connection	Dashed arrow
Fixed connection	Solid line

A flexible connection between a link and a joint describes the effect of the joint on the connected link, that is, along with the specification of the joint, it describes how the connected link can move relative to the joint’s containing link; a flexible connection is represented by a dashed line. In Fig. 2, the joints LHinge and RHinge contained in the link Core have flexible connections to the links called Wheel in the parts LTreel and RTreel.

A connection between two links via a solid line defines a fixed connection. In Fig. 2, there are fixed connections between the Core and the links Track in LTreel and RTreel. So, these links are attached, but there is no joint between them.

#### p-models

In RoboSim, a block diagram defines a single p-model consisting of a p-model block, and all the blocks are connected to it, directly or indirectly, via containment, flexible, or fixed connections. The p-model block is connected to other blocks defining the elements contained in the platform or part modelled by the diagram. Figure 2, for instance, contains a single p-model block BaseModule for the robotic platform in our example.

In every diagram, the containment relationship defines a tree, where the element represented by a parent block contains (or is composed of) the elements represented by the children blocks. The root is the unique p-model block.

***Links and bodies***

A link is a representation of a rigid component, that is, no motion is possible between the bodies that define the link’s geometry. Motion is only possible between different links and is specified by joints. Links are used in the construction of the model for a platform or part, and may contain joints, sensors, actuators, and bodies. A link cannot contain other links or parts.

A body captures the physical properties of a link, but cannot contain joints, sensors, or actuators. (Bodies, sensors, and actuators cannot contain any other elements.) We cannot define connections to or from bodies. Since a body is a property of a link, it gives a partial view of a component. It is not a component that can be considered in its own right (and therefore, be connected to, or contain, others).

**Fig. 4:**
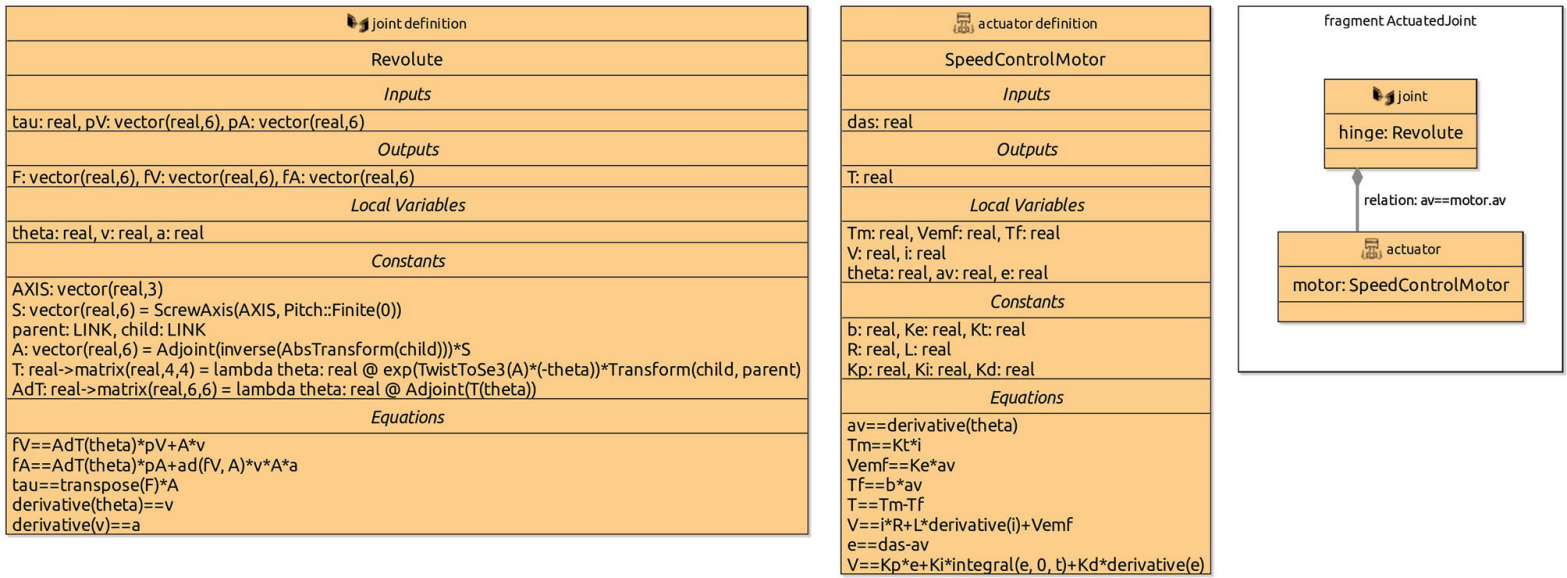
Library elements

***Joints***

Two links can also be connected via joints. In this case, the joint is defined to be contained in one of the links, and to have a flexible connection to the other. In Fig. 2, Core contains two joints LHinge and RHinge of a Revolute type defined in the RoboSim library. These joints have flexible connections to the Wheel links in LWheel and RWheel. Movement of a joint induces movement on the link to which it is flexibly connected.

The RoboSim library contains a collection of widely used joint definitions. It is possible, however, to define a customised joint, by defining its behaviour using differential equations, like it is done for the library joints. The equations relate inputs and outputs of the joint, possibly in terms of local variables and constants. (Similarly, we can specify the behaviour of sensors and actuators.)

To illustrate how the behaviour of a joint can be specified, we present in Fig. 4 the library definition for Revolute. This block characterises a joint definition, that is, a design, rather than a specific realisation of that design. (This is indicated in the block by the term joint definition at the top next to the icon.) In the BaseModule p-model in Fig. 2, LHinge and RHinge are two different realisations of that design as said above.

The definition of Revolute indicates that it takes as input the torque tau, and two vectors pV and pA of velocities and accelerations of the link that contains the joint. These vectors contain both angular and linear components. The joint outputs three vectors F, fV and fA of forces and torques, velocities, and accelerations induced on the link flexibly connected to the joint. The equations that relate these inputs and outputs are based on a restructuring of the screw theory equations of motion discussed in [46]. They use constants also declared in the Revolute block and functions. Standard functions, such as ScrewAxis, are defined in the RoboSim library, but functions can also be defined as part of a model; we omit the definitions of functions in the library here.

**Fig. 5:**
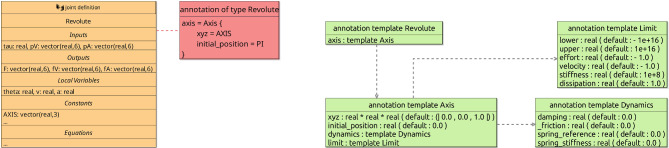
Annotation and annotation templates

The values of the constants may or may not be defined. For example, in Fig. 4, the value of the constant AXIS in the block Revolute is not defined. In a realisation, it is possible to define a value, as illustrated in Fig. 2 where AXIS is given value (0,1,0) in both LHinge and RHinge.

Joints can contain sensors and actuators. In Fig. 2, the joints LHinge and RHinge contain motors specified by the actuator blocks LMotor and RMotor further discussed next.

***Sensors and actuators***

Sensors and actuators are components used to, respectively, obtain information from the environment and produce an effect on the environment; they are the primary means of interaction between a robot and its environment. The physical behaviours of sensors and actuators can be defined using differential equations. In Fig. 2, for example, we have Proximity sensors, which are realisations of an infrared sensor definition IR in the RoboSim library (omitted here). In the definition of IR, we have a single equation. The input is the distance between the sensor and an obstacle in the environment. That input can be provided, for example, by a model of the environment (in a simulation). The output is a voltage that reflects that input. The equation that relates voltage and distance can be obtained, for example, by curve fitting based on experiments with the sensor.

The motors LMotor and RMotor in Fig. 2 are realisations of the actuator definition SpeedControlMotor. This block is part of the library, as indicated in Fig. 4. In SpeedControlMotor, we specify the desired angular speed das as input, and the torque T produced as output. The system of equations is an adaptation of the model in [49]. The variables are the motor torque Tm, the back electromotive force Vemf, the torque due to viscous damping Tf, the voltage V supplied to the motor by the embedded controller, the current i, and the angular position theta, speed av, and speed error e. The constants include the viscous damping b of the motor, the electromotive force constant Ke, the motor torque constant Kt, the electric resistance R, the electric inductance L, and the PID constants Kp, Ki and Kd.

The values of the constants are neither defined in SpeedControlMotor nor in its realisations LMotor and RMotor. So, although used just to define the behaviour of the motors, these are constants of BaseModule as a whole. Because they are declared locally in a block, though, they can only be used in that block. In general, constants whose values are not determined in the model are implicit parameters of that model. When generating a simulation or carrying out a verification by model checking, the values of these constants need to be defined. They can be, for example, subject to design-space exploration to define, via simulation perhaps, the values that best fit the application.

If an actuator affects the robotic platform or part defined by the p-model itself, or, similarly, if a sensor collects information about elements of the p-model, it is necessary to capture this relationship by labelling the containment relationship with an equality. In Fig. 2, the input tau for LHinge is equated to the output T of the actuator LMotor, and similarly for RHinge and RMotor. This captures the fact that the output of the motor affects the behaviour of the joint.

#### Structuring mechanisms

***Parts***

A block diagram that defines a part, rather than a complete platform, also contains a block that specifies the p-model for the part. The possibility to define p-models for parts allows us to define for reuse particular combinations of links, joints, sensors, actuators, and bodies, possibly via the use of parts defined themselves by further diagrams. Via a library of p-models for parts, as well as of pre-defined links, joints, sensors, actuators, and bodies, we can extend the RoboSim notation to suit the needs of particular areas of application.

**Fig. 6:**
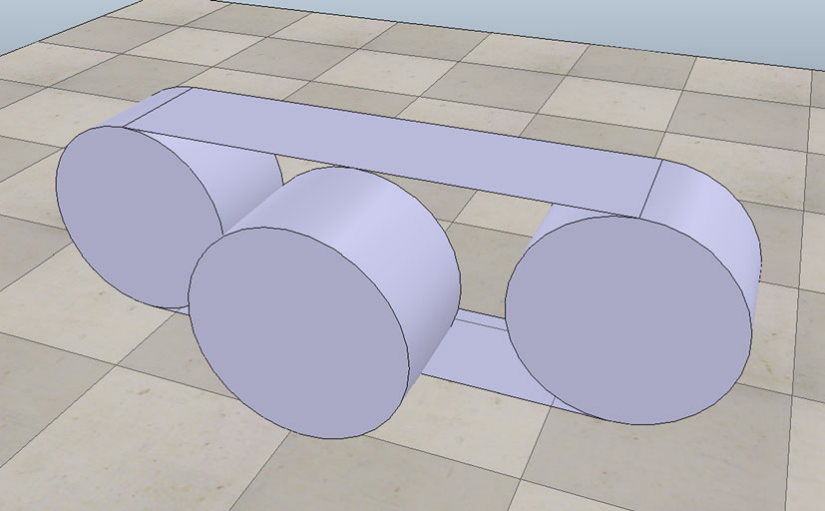
Rendering of top view of Treel produced by CoppeliaSim

**Fig. 7:**
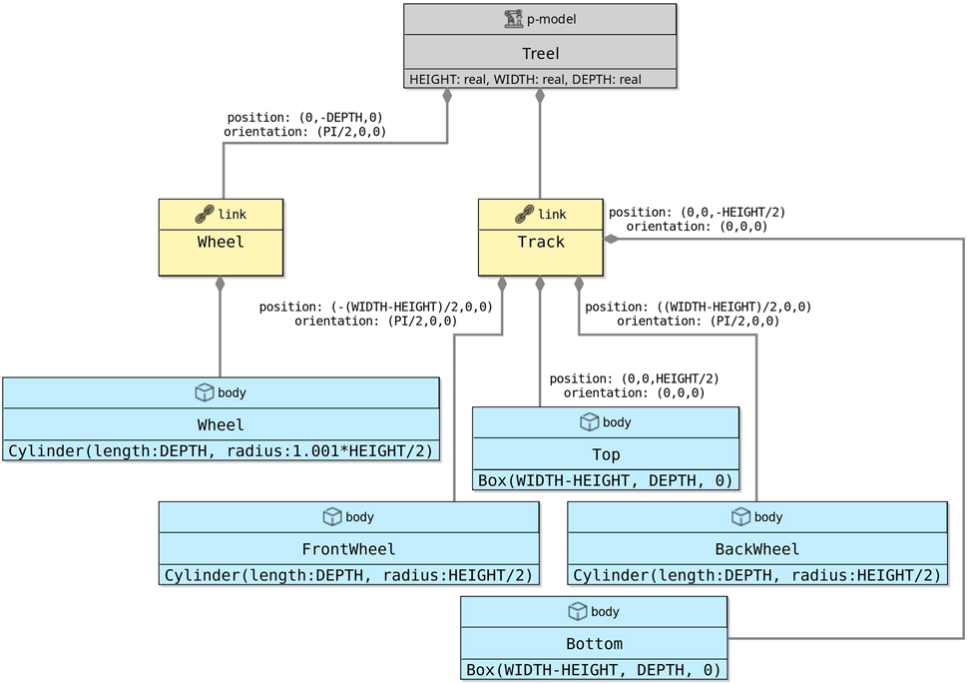
p-model of a treel (track + wheel)

A p-model for the part in Fig. 6 (used in Fig. 2), containing a track and three wheels, is shown in Fig. 7. This p-model Treel contains links Track and Wheel, each with a body.

A p-model block (for a part or platform) can also declare constants for use in the definition of attributes of other blocks in the diagram. In Treel, there are constants for the HEIGHT, WIDTH, and DEPTH of the Track, also used to define the length and radius of the Wheel body, which is a Cylinder. The values of such constants can be fixed in a p-model, but, like in our example, can be left open like for any other constant defined in the blocks.

If a connection is to or from a part, whose links and joints are therefore defined in a separate block diagram, the link or joint inside the p-model for the part that is really connected must be identified. This is done by annotating the part-side of the connection with the name of the link or joint in the part. For example, the connections between the revolute joints LHinge and RHinge in Fig. 2 and the parts LTreel and RTreel are labelled with the connecting link Wheel of the part (defined in the diagram for Treel in Fig. 7). Connections between two parts are labelled on both sides.

***Frames of reference and poses***

A p-model block has position (0,0,0) and orientation (0,0,0) as its (implicit) frame of reference, used to define the pose of the elements in the p-model by labelling their containment connections.

A pose determines a position via *x*, *y*, and *z* coordinates, and an orientation, that is, roll, pitch, and yaw, all using the SI units. For example, in Fig. 7, the Track of the Treel is positioned so that it is displaced on the z-axis by half of its overall HEIGHT, but has the same orientation of the Treel. The third Wheel is displaced by half of its depth and turned on its side (see Fig. 6). Although we adopt SDF and URDF conventions, such as the use of roll-pitch-yaw Euler angles, extending our tools and techniques to support modelling using alternative conventions is relatively simple (with only minimal and localised impact on the metamodel and model transformation techniques presented in the sequel). Nevertheless, the use of a different representation may create difficulties in the interpretation of the analysis and simulation of the models.

Going down the containment hierarchy, the position and orientation of the Track block implicitly determine a frame of reference for the blocks that are contained in it. The position and orientation of its bodies (Top, FrontWheel, BackWheel, and Bottom) are defined with respect to that implicit frame of reference. In general, throughout the hierarchy of the containment relationship, the pose of a child block is defined in relation to the frame of the parent block as a label of the containment connection. (If the child block has the same pose, the label can be omitted for simplicity.)

When Treel is used to create the parts LTreel and RTreel in Fig. 2, their poses are specified in the containment relationship. The orientation of RTreel is unchanged, but LTreel is rotated PI radians around the x-axis. So, the Wheel is always facing outwards. The parts are also translated up (both 2.5cm on the z-axis) and sideways (-3 cm and 3 cm on the y-axis). The overall effect is the composition of the two poses, that is, the pose of Wheel with respect to Treel, and the pose of LTreel and RTreel with respect to BaseModule, with the innermost pose applied first (see Fig. 3).

***Multiplicities***

If a p-model contains several elements of the same kind, for instance, several sensors with the same properties, we can use indexation when defining containment. For example, the BaseModule in Fig. 2 contains 24 IR sensors distributed around its Core. The containment relationship between these blocks, therefore, has an index i, whose values vary between 0 and 23 as defined in the declaration of i. As shown, the index can be used to define the position and orientation of each element. It can also be used in the definition of the equations, if any, of the contained block.

***Library and fragments***

As we have said before, the RoboSim library includes definitions for joints, sensors, actuators, and p-models (for parts). It is also possible to include in the library model fragments, composed of several blocks, but not defining a (well-formed) p-model. When a fragment is used in a p-model, all its blocks and connections are directly included. This is in contrast with the use of a p-model, which, we recall, is via a part block that declares a name for a part defined by that p-model.

For example, Fig. 4 shows a fragment ActuatedJoint, which describes an electrically actuated joint formed by a revolute joint and a motor. This fragment does not define a physical model, since a joint does not have a body. It is also not a valid p-model, where joints cannot occur disconnected. The fragment, however, is useful to define, for example, the p-model in Fig. 2.

#### Platform mappings

**Fig. 8:**
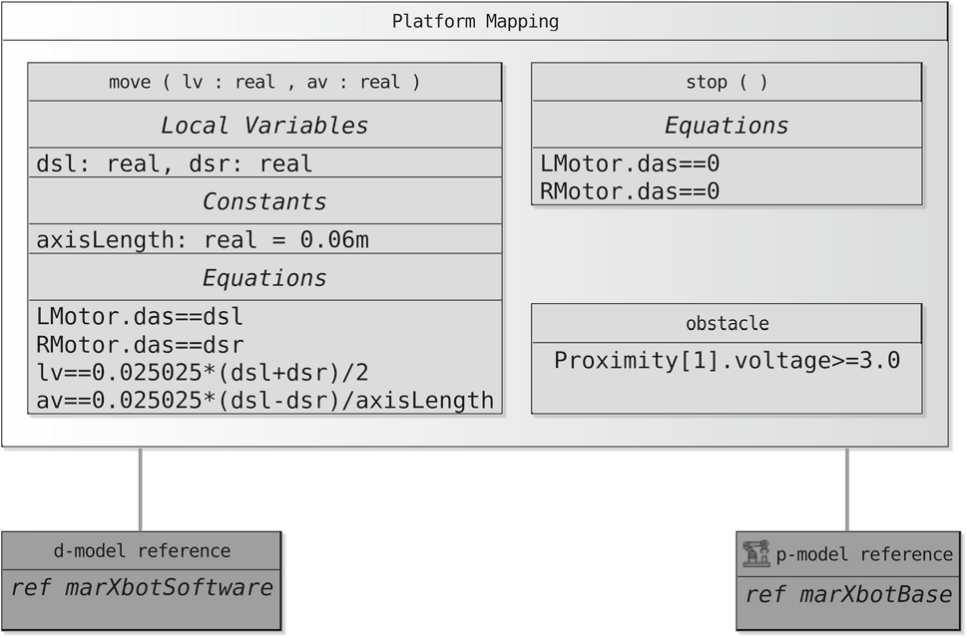
Property of the connection between SimCMovement and the block diagram for marXbot

As mentioned, the association between the d-model and the p-model of a robotic platform must define how the variables, events, and operations of the d-model are realised by elements of the p-model. In Fig. 8, references to the RoboSim module (d-model) in Fig. 1 and to the p-model in Fig. 2 are connected via a platform mapping block, where three inner mapping blocks define the event and operations of the d-model.

**Fig. 9:**
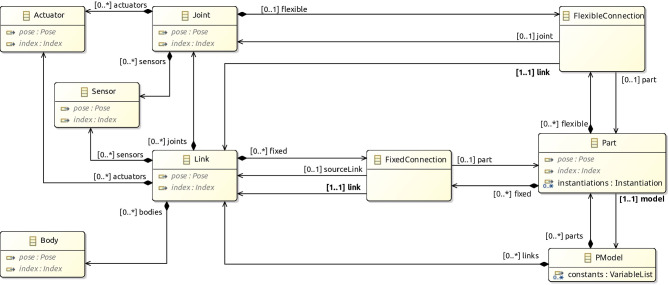
Main components of the metamodel for block diagrams for a RoboSim p-model

Mappings for input events have two components. The first is a predicate that defines when the event occurs in terms of sensor outputs. In our example, obstacle is an input event defined by a predicate that determines that an obstacle is present when the voltage output by the Proximity sensor of index 1 is greater than or equal to 3.0. If the input event communicates values to the software, these values are defined in the second component of the mapping. This is an action that assigns, to the inputs, values determined by an expression involving the outputs of the sensors.

Whether an output event occurs or not is determined by the software. If it does, the event mapping includes an action that assigns, to inputs of the actuators, values that may depend on communicated outputs. So, a mapping for an output event defines its effect on the actuators.

The operation mappings are defined by actions or differential equations. An action may assign directly to inputs of actuators. Assigning expressions typically depend on the parameters of the operation, if any. In our example, stop() is defined by assigning 0 to both LMotor.das and RMotor.das. The move(lv,av) mapping is defined by four equations that relate the inputs LMotor.das and RMotor.das of the motors, and the parameters lv and av of move(lv,av). For conciseness, the equations can use local variables and constants. In our example, these are dsl, dsr, and axisLength.

Like output events and operations, variables of a d-model are abstractions for inputs to actuators. So, a mapping block for a variable assigns, to an input of an actuator of the p-model, an expression involving that variable and, possibly, configuration variables and constants of the actuator.

#### Annotations

Joints, sensors, and actuators can be annotated with extra information (typically, to inform their translation to SDF or another domain-specific language). In Fig. 5, we give an example of an annotation for Revolute, as indicated by a dashed line connecting the Revolute block to an annotation block. This joint is that in Fig. 4, partly elided for conciseness. Annotations play no role in the mathematical (*CyPhyCircus*) semantics of the p-model. They are, however, useful to improve the automation of the simulation generation.

As another example, we observe that the equation defining the sensor IR does not identify a particular sensor. The equation is a general characterisation of the expected sensor behaviour, which can be realised by a sonar, a lidar, or a camera, for example. Since SDF does not cater for equational definitions, an annotation is useful to indicate the kind of sensor that should be used in an SDF document. Later in Sect. 5, we describe our approach to translation to SDF.

Annotations instantiate a template. In Fig. 5, the annotation for Revolute is an instance of the template also called Revolute (although these names do not need to match) in Fig. 5. An annotation template defines the parameters whose values can be specified in an annotation that instantiates that template. For example, a Revolute annotation has a single parameter axis, which is an instance of another template Axis. Dashed arrows indicate that the template at the source uses the template at the target. A template can optionally provide default values for the parameters, which can be overridden in actual annotations.

In an annotation, we can use the constants and variables of the annotated element. For example, in the annotation for Revolute in Fig. 5, the value of the xyz parameter is given by the constant AXIS of Revolute. The axis parameter in the annotation records information about the axis in the parameters xyz, initial_position, dynamics, and limit as defined in the template Axis in Fig. 5. For example, it states that the axis has initial_position PI, but the values of limit and dynamic are the default given in the template. So, limit and dynamic are omitted in the instantiation. The constant PI is defined in the library.

This annotation, and others in the RoboSim library, reflect information that is required in an SDF document to specify a revolute joint. That library is used in our translation from RoboSim to SDF (see Sect. 5). The possibility of defining templates, however, means that annotations are not fundamentally tied to SDF. It is possible, for example, to create and use another library of annotation templates tailored to URDF, or to the needs of a particular simulator.

Next, we describe the metamodel of p-models.

### Metamodel

**Fig. 10:**
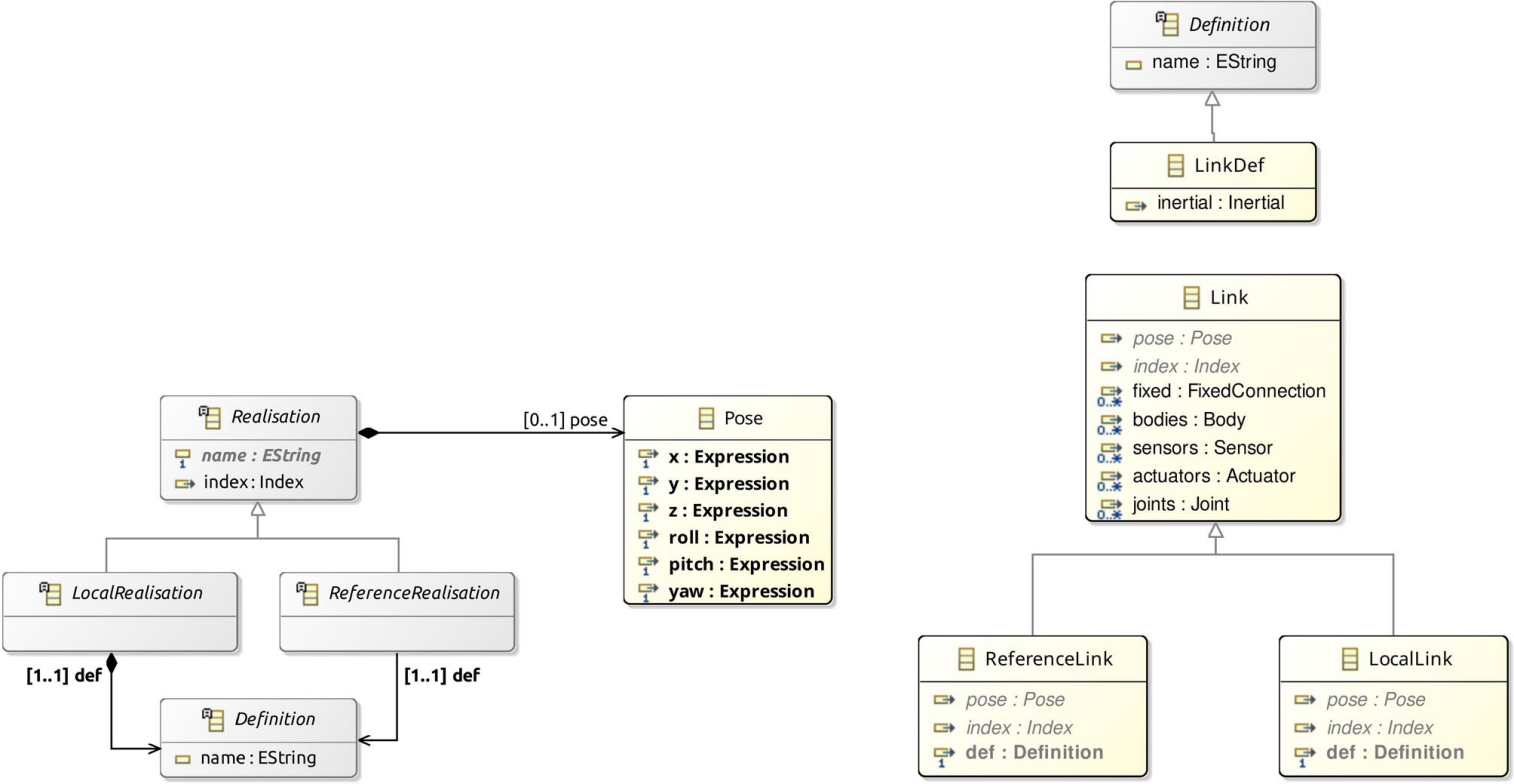
Metamodel for Link blocks

**Fig. 11:**
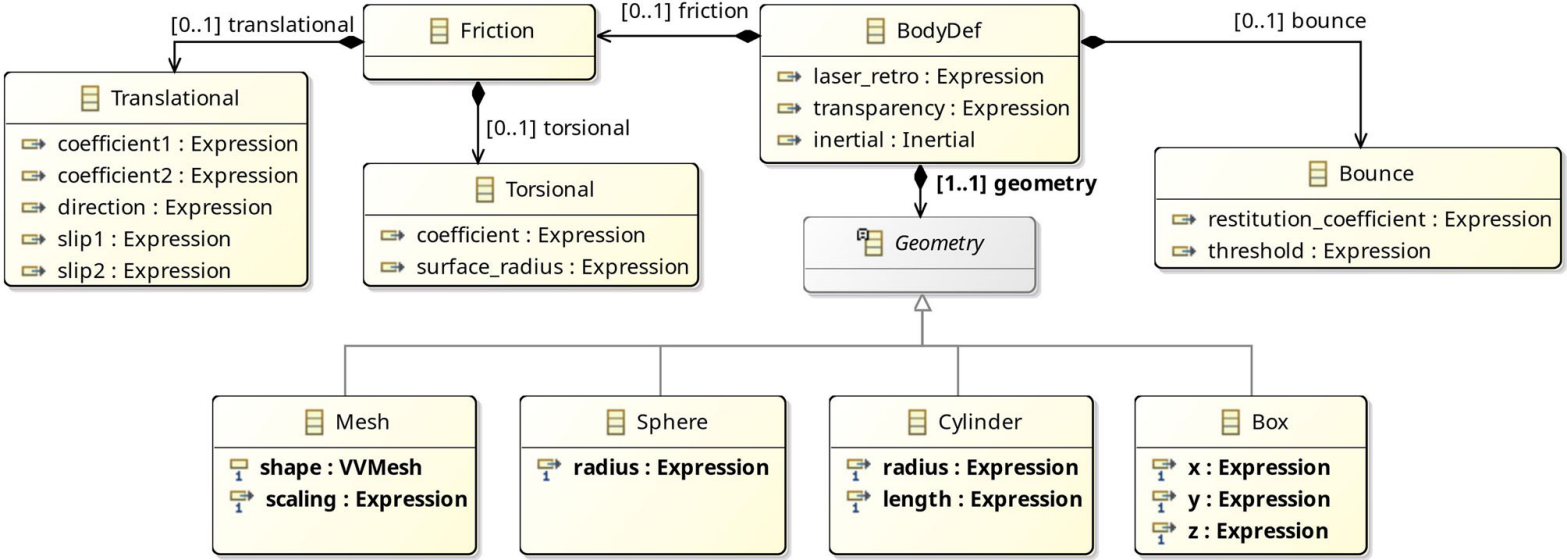
Metamodel for Body blocks

**Fig. 12:**
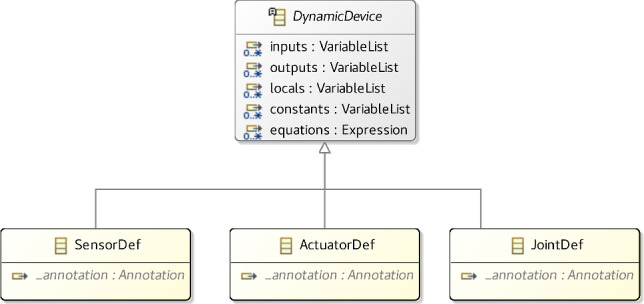
Metamodel for DynamicDevice

**Fig. 13:**
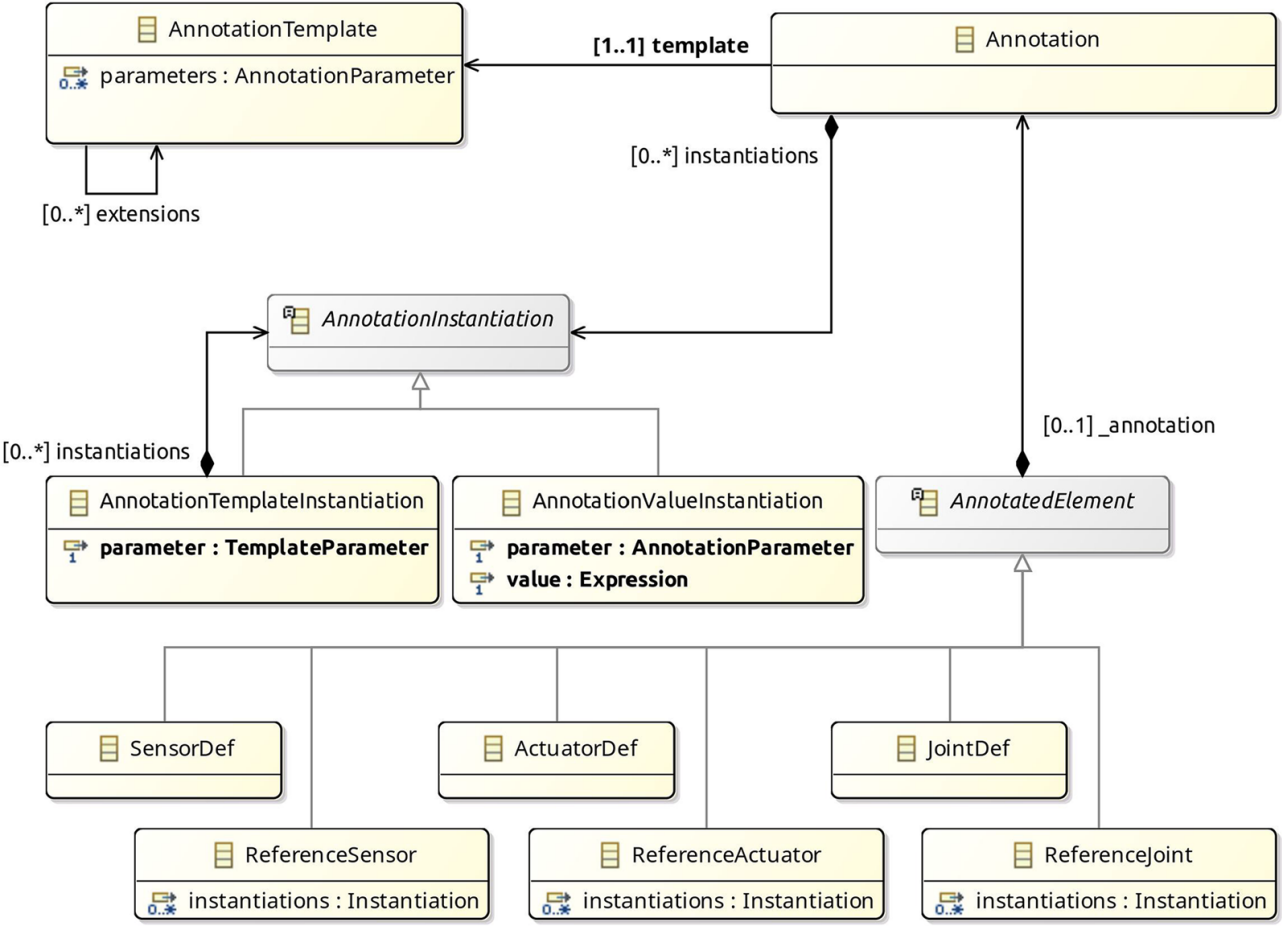
Metamodel for Annotation

**Fig. 14:**
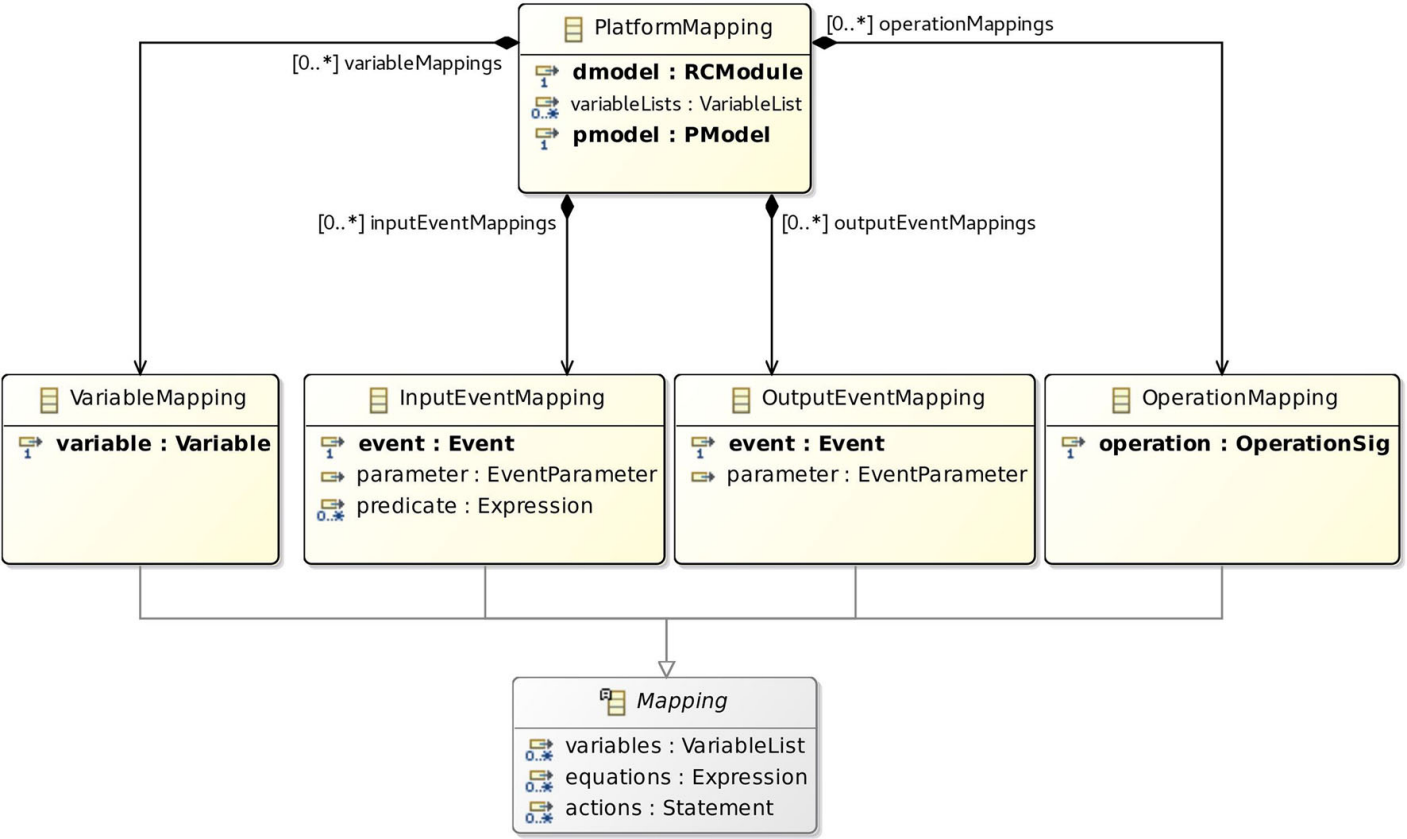
Metamodel for PlatformMapping

Figure 9 gives an overview of the metamodel of a RoboSim block diagram. Further details are provided in Figs. 10-14. The main element of a p-model is an object of the class PModel. It defines any number of parts, links, and constants, and the latter listed as an attribute in the PModel block.

A Link describes a physical realisation, as opposed to a definition, of a link. It contains information specific to its use in the artefact represented by the PModel. For example, a pose is an attribute of Link. In contrast, a link definition can be realised in several diagrams or several times in a diagram, with different poses. Each realisation is represented by a different Link, with its own fixed connections, bodies, sensors, actuators, and joints.

Figure 10 gives more details. Link is a subclass of the abstract class Realisation shown on the left. (The inheritance relationship between Link and Realisation is omitted in Fig. 10.) Other subclasses of Realisation, not shown in Fig. 10, include Joint, Sensor, Actuator, Part, and Body.

Realisation has an (inherited) attribute name, optional attributes pose and index, and two abstract subclasses. A Realisation can be either a ReferenceRealisation, described via a reference to a Definition via the attribute def, or a LocalRealisation, including the definition itself, in an attribute also called def, but that composes the LocalRealisation. A Definition is an element with a name. Realisation and its two abstract subclasses ReferenceRealisation and LocalRealisation define a mechanism by which we can specify a physical element in a p-model either via a reference to an independent (library) Definition (that is, an object of class ReferenceRealisation) or via a local Definition (that is, a LocalRealisation).

Link inherits from Realisation the attributes name, pose, and index. The Link subclasses named ReferenceLink and LocalLink are as expected subclasses of ReferenceRealisation and LocalRealisation (but this is not shown in Fig. 10.) For a ReferenceLink and a LocalLink, the definition is a LinkDefinition (omitted in Fig. 10), which encapsulates inertial information. This is enforced by well-formedness conditions in Sect. 4.3. LinkDefinition is a subclass of Definition.

Figure 9 also shows that a Link can contain fixed connections of class FixedConnection. Each connection is to another link. A well-formedness condition ensures that a link is not connected to itself. If the FixedConnection is contained in a Part, rather than a Link, the extra attribute sourceLink identifies the link in the Part being connected. The connection can also be to a Part identified by the attribute part, rather than directly to a link. In this case, link identifies a link in the Part model.

A Part identifies a p-model, that is, an object of class PModel itself. It includes instantiations of any constants of the referenced PModel. For example, in Fig. 2, the Part that introduces LTreel instantiates the constants HEIGHT, DEPTH, and WIDTH of the p-model Treel in Fig. 7.

The metamodel for Joint is similar to that for Link in Fig. 10; a Joint is a realisation of a JoinDefinition. A Joint can contain a flexible connection, of class FlexibleConnection. The attributes of FlexibleConnection include a link. Even if the FlexibleConnection is to a part, the connection identifies a link in the model for the part. The connection is associated with the joint that contains it. A FlexibleConnection can also be contained in a Part. In this case, the attribute joint of the connection identifies a joint in that part.

A Link and a Joint can contain actuators and sensors. Like Link and Joint, the classes Actuator, Sensor, and Body represent realisations of an ActuatorDefinition, a SensorDefinition, or a BodyDefinition. The metamodels for Actuator and Sensor are similar to that for Link in Fig. 10.

The metamodel of Body is also similar to the metamodel of Link, but the definition of a body (BodyDef) includes extra properties specific to rigid bodies, such as friction, bounce, and geometry. These properties are not included in the metamodel for joints, sensors, and actuators because they are specified explicitly via a set of equations, which can be directly enriched to model aspects such as damping and friction.

For example, the equations of the Revolute joint in Fig. 4 can be extended to account for damping and friction. Damping can be modelled as in the SpeedControlMotor via a damping constant b, which is used to define a torque taub via the equation taub = -b*v. Friction can be included by considering friction models of shaft joints that incorporate the joint axial force (fFN), the joint radius (fR), and the friction coefficient (mu), which are assumed to be constants and used to estimate the opposing frictional torque taufd as follows.

taufd = mu*fFn*fR*(sgn(v)) + b*v

Here, sgn is a function that calculates the sign of its input. Finally, the equation that defines the total torque tau can be modified as follows.

tau==transpose(F)*A-taufd

This flexibility allows the development of libraries of definitions targeting a variety of approaches, such as those in [46] and [25, 26].

On the other hand, SensorDef, ActuatorDef, and JointDef, besides inheriting from Definition, inherits also from DynamicDevice as shown in Fig. 12. A DynamicDevice can have its behaviour specified by equations that relate inputs and outputs potentially using local variables (locals) and constants (recall Fig. 4).

VariableList represents the declaration of one or more variables, which may have a modifier to indicate that they are constants. This class is part of the d-model metamodel [20]. Equally, the Expression language is similar to that of RoboSim d-models, but includes derivatives, integrals, and the possibility to define SI base and derived units.

The metamodel for Annotations is in Fig. 13. An Annotation always refers to a template, providing instantiations for its parameters. An AnnotationTemplate defines parameters directly or via extensions of other AnnotationTemplates. A parameter is an object of the class AnnotationParameter (omitted in Fig. 13). Examples of parameters are given in Fig. 5 on the right; for instance, axis is a parameter of Revolute. Its declaration gives its name and type. In some cases, we give a default value; see, for instance, xyz in Axis.

An AnnotationInstantiation can be either an AnnotationValueInstantiation or an AnnotationTemplateInstantiation. The former simply identifies a parameter and defines a value for it. An AnnotationTemplateInstantiation defines a collection of instantiations for a parameter whose type is a template: this is a TemplateParameter. For example, in Fig. 5, axis is a TemplateParameter of Revolute. Its instantiation on the left of Fig. 5 defines instantiations for xyz and initial_position.

An Annotation can be optionally included in any AnnotatedElement. This is an abstract class whose concrete subclasses include definitions and references to sensors actuators and joints.

The metamodel for a PlatformMapping is shown in Fig. 14. It relates a dmodel (of class RCModule of the d-model metamodel) to a pmodel, and might declare variables for common use of the Mappings. A PlatformMapping can have collections of variableMappings, inputEventMappings, outputEventMappings, and operationMappings. They include instances of the appropriate subclass of the abstract class Mapping.

In any Mapping, we can declare local variables, and use equations or actions to define a variable, event, or operation. The concrete subclasses of Mapping identify one such element; for instance, an OperationMapping identifies the operation that it defines. The class OperationSig is from the d-model metamodel. In addition, an event may have a parameter representing the values it communicates. This is taken into account in InputEventMapping and OutputEventMapping. Finally, a predicate indicates when and input event happens. Event, EventParameter, and Statement are from the d-model metamodel.

Full details of the metamodel, including the classes omitted here, are in [51].

### Well-formedness conditions

Not every object of a metamodel class represents a valid component of a RoboSim block diagram. For example, a p-model with a joint that is (flexibly) connected to a link that is not contained in that p-model does not represent a valid physical artefact. We have specified 39 well-formedness conditions that rule out such p-models. They identify the valid p-models that can be translated to SDF and given a mathematical description. Many conditions are expected of a standard block diagram notation, and include well-typedness and obvious scope rules. Our additional 39 conditions are specific to RoboSim or related to constructs specific to RoboSim. We note, however, that issues related to physical restrictions, such as overlapping bodies and pose compatibility, are left to be checked by simulators and physics engines.

We describe and justify all our conditions below. The complete set is in [51].

***p-model***

Only one condition applies to a p-model.

*PM1*: *A* PModel * has to contain at least one part or link, that is, at least one of the attributes* parts * or* links * has to be non-empty.*

Without links or parts, there can be no physical presence, or even an abstraction of a physical presence. As already explained, joints have to be contained in a link, and sensors and actuators have to be contained in a link or joint. So, parts and links are the key elements to be included.

***Links and bodies***

As mentioned previously, we need well-formedness conditions to ensure that the right type of Definition is associated with each type of Realisation. For links, these well-formedness conditions are below.

*L1*: *The value of* def * of a* LinkReference * must be of type* LinkDef .
*L2*: *The value of* def * of a* LocalLink * must be of type* LinkDef .

In addition, Definitions specified for reuse must have a name, but those included in a p-model must not have a name, since the LocalRealisation that includes the Definition already has a name. For a link, the well-formedness condition is as follows.

*L3*: *A* LinkDef * defined directly in a package must have a name, while a* LinkDef * defined as part of a* LocalLink * must not have a name.*

Similar restrictions apply to all forms of Definition, that is, JointDef, SensorDef, ActuatorDef, and BodyDef. We omit these well-formedness conditions here, but they are all listed in [51].

Finally, the inertial for a link can be recorded directly in its definition or in its bodies. It should not be provided in both, though.

*L8*: *If the* inertial * attribute is defined in a* LinkDef * l, then none of the* bodies * for a realisation of l, can define* inertial * as well.*

This ensures that no conflicting or duplicated inertial information is recorded.

***Joints***

We have the following extra condition for joints.

*JSA1*: *The* relation * in a* Sensor * or in an* Actuator * is either an equality or a conjunction of equalities*.

The equalities define the inputs of the sensor or how the outputs of the actuator are used.

***Fixed and flexible connections***

We have similar well-formedness conditions for fixed (Fix1–3) and flexible (Flex1–3) connections. Collectively, they ensure that connections are between links and joints of the same p-model. These may be directly included in the p-model, or links and joints of parts contained in the p-model. We list here the conditions for FixedConnections.

*Fix1*: *A* fixed * connection of a* Link * l is either to a* link * (different than l) contained in the p-model containing l or in the p-*model * that defines a* Part * contained in the p-model containing l.*
*Fix2*: *A* fixed * connection of a* Part * p must have a* sourceLink * contained in the p-*model * that defines p.*
*Fix3*: *A* fixed * connection of a* Part * p is either to a* link * contained in the p-model containing p or in the p-*model * that defines a* Part * contained in the p-model containing p.*

The conditions for FlexibleConnection are in [51].

***Parts***

A part is an instantiation of a p-model in the context of another. In the instantiation, all constants declared in the part’s p-model must be given a value (P1), if they do not have one already (P2).

*P1*: *A part must instantiate all uninitialised constants of the p-*model * that defines it*.
*P2*: *A part must not instantiate initialised constants of the p-*model * that defines it*.

Of course, we can define values using constants of the p-model that uses the parts.

***Annotations***

For annotations, the well-formedness conditions (omitted here) just ensure that AnnotationTemplates define a hierarchical model using the extensions mechanism, and that the Annotations are well-typed instances of these templates.

***Platform mappings***

A platform mapping is defined for a p-model for a complete robotic platform, not just a part. There are three ways in which elements of a p-model can be connected: via containment, or via fixed or flexible connections. Containments and fixed connections are optional, but, for a joint of a complete p-model, the flexible connection is not optional. So, we have the following restriction.

*PMap1*: *In the* pmodel *, every* Joint * contained in a link of that* PModel * has a* flexible * connection.*

Unlike a part, a robotic platform is a complete artefact. So, in its p-model every joint should be used to connect two links. In contrast, in a p-model for a part, there is no such restriction.

A similar well-formedness condition considers the joints in the parts of the p-model. The next two conditions ensure that the d-model and p-model are properly matched by the mappings.

*PMap3*: *There is a* Mapping * for every event and operation of the* dmodel *.*

This ensures that the abstractions of the d-model are realised by the p-model. In the case of variables, the presence of a mapping is not enforced. Variables without such a mapping are part of a shared memory available to controllers, and are not realised by the p-model.

*PMap4*: *Every* Mapping * is for a variable, event, or operation of the* dmodel .

So, the d-model establishes a scope for the platform mapping. Moreover, the presence or otherwise of a type definition for an event determines whether a communicated value can be defined.

*PMap5*: *If, and only if, the* event * of an* InputEventMapping * or* OutputEventMapping * is typed in the* dmodel *, that* InputEventMapping * or* OutputEventMapping * declares a parameter, which must be of that same type.*

In this way, its equations or actions can use or define the value communicated via the event.

Next, we ensure unicity of definitions.

*PMap6*: *In a* Mapping *, there are* actions * or* equations *, at least one, but not both.*

These are mutually exclusive forms of specifying how the d-model components are realised.

Additional conditions ensure that the Mappings establish the expected connections between the d-model and the p-model. For instance, the condition for an InputEventMapping is as follows.

*PMap7*: *The* equations * or* actions * in an* InputEventMapping * can read from the outputs of sensors, local variables and constants, and it can write to local* variables *, and, if the* event * is typed, its parameter.*

This reflects the fact that an input event is an abstract representation of a sensor.

Similar conditions restrict the read and write accesses of the other Mappings, taking into account that output events, operations, and variables are abstractions for actuators (instead of sensors).

*PMap11*: *The* actions * cannot call* dmodel * operations.*

This reflects the fact that operations of the d-model should, in as far as it is possible, correspond to independent abstractions for facilities to control the robot available in the platform. If two operations are called in the same cycle of simulation, for example, their behaviours should not conflict. For example, we should not call operations to move and stop the robot in the same cycle of simulation.

The well-formedness conditions have a role in eliminating invalid models from consideration when generating simulations and mathematical models. They also have a role in providing modelling guidance. For example, we can handle (that is, generate an SDF document and *CyPhyCircus* for) a p-model that does not satisfy *PMap*1. This well-formedness condition helps the identification of mistakes when the p-model is intended to define a complete artefact, not a part. Another example is *L*4, which indicates how to capture inertial information. Use of XML-based notations like SDF, for instance, does not provide this sort of guidance. Next, we compare and relate RoboSim and SDF.

## Mapping to SDF

In this section, we first give a brief overview of SDF in Sect. 5.1, and then contrast the features of RoboSim block diagrams and SDF in Sect. 5.2. Finally, in Sect. 5.3, we present our model transformation technique to translate (well-formed) RoboSim block diagrams to SDF documents.

### SDF

**Fig. 15:**
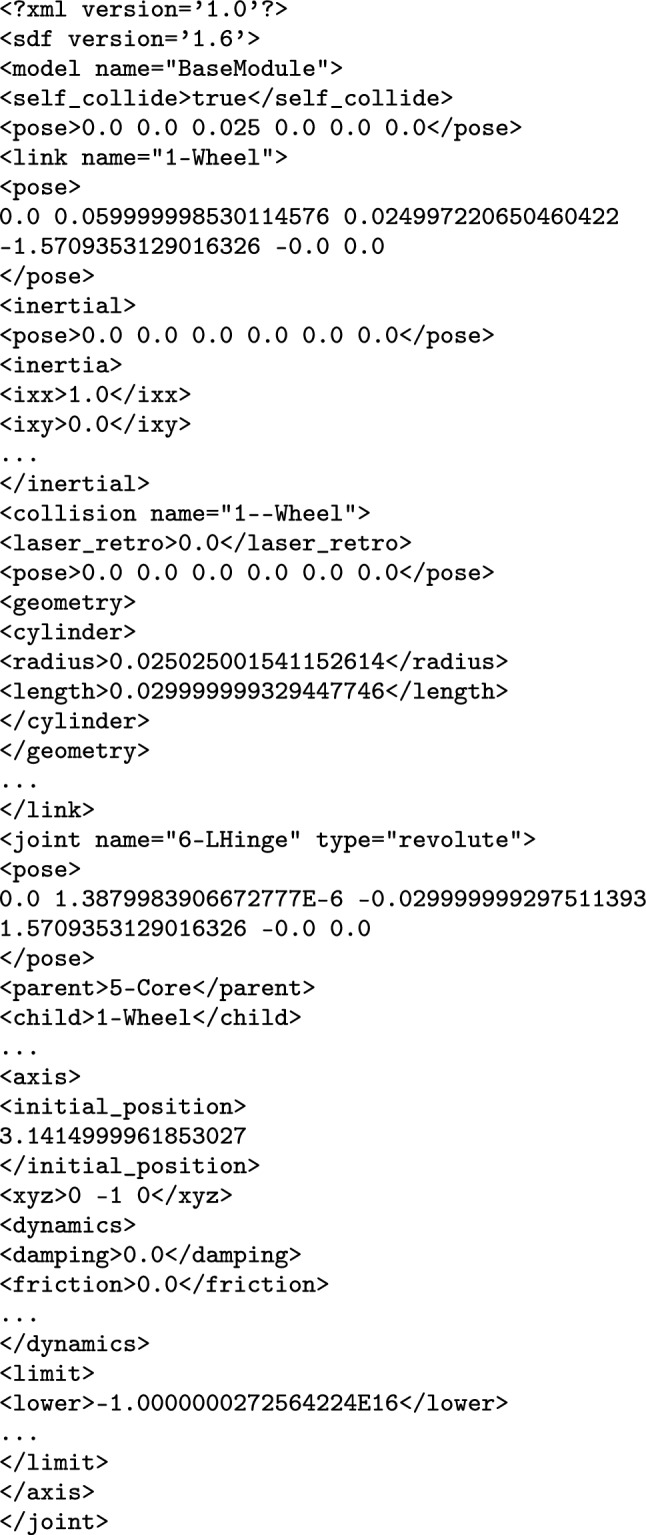
Sketch of SDF document for BaseModule in Fig. 2 that is automatically generated by RoboTool

SDF is an XML format to describe elements such as robotic platforms, physical objects, and environments for robotic simulators. SDF embeds domain knowledge via XML tags used to describe elements and their attributes; the root element is described by the tag $\texttt {<}$sdf$\texttt {>}$. Figure 15 shows an excerpt of the SDF specification for the robotic platform on the right in Fig. 3. The complete document has 1322 lines and is available.^13^

Each element may have zero or more optional or mandatory child attributes and elements. Table 3 exemplifies some of the elements of the SDF format that can be used for specifying a robotic platform. The $\texttt {<}$sdf$\texttt {>}$ element has a child attribute, called version, which describes the version number of the SDF format used. The $\texttt {<}$sdf$\texttt {>}$ element can also have a child element tagged $\texttt {<}$model$\texttt {>}$, which specifies a physical object or robot. A $\texttt {<}$model$\texttt {>}$ tag requires the definition of a name. As an example, the name of the model element in Fig. 15 is “BaseModule".

**Table 3:** Some of the elements and attributes of the SDF format that can be used for specifying a robotic platform

Element	Child attribute	Child element
$\texttt {<}$sdf$\texttt {>}$	version	$\texttt {<}$model$\texttt {>}$
$\texttt {<}$model$\texttt {>}$	name	$\texttt {<}$pose$\texttt {>}$, $\texttt {<}$link$\texttt {>}$, $\texttt {<}$joint$\texttt {>}$
$\texttt {<}$link$\texttt {>}$	name	$\texttt {<}$pose$\texttt {>}$, $\texttt {<}$collision$\texttt {>}$, $\texttt {<}$visual$\texttt {>}$, $\texttt {<}$sensor$\texttt {>}$
$\texttt {<}$collision$\texttt {>}$	name	$\texttt {<}$pose$\texttt {>}$, $\texttt {<}$geometry$\texttt {>}$, $\texttt {<}$laser_retro$\texttt {>}$, $\texttt {<}$surface$\texttt {>}$
$\texttt {<}$geometry$\texttt {>}$		$\texttt {<}$box$\texttt {>}$, $\texttt {<}$cylinder$\texttt {>}$, $\texttt {<}$sphere$\texttt {>}$, $\texttt {<}$mesh$\texttt {>}$
$\texttt {<}$sensor$\texttt {>}$	name, type	$\texttt {<}$pose$\texttt {>}$, $\texttt {<}$camera$\texttt {>}$, $\texttt {<}$gps$\texttt {>}$, $\texttt {<}$ray$\texttt {>}$, $\texttt {<}$sonar$\texttt {>}$
$\texttt {<}$joint$\texttt {>}$	name, type	$\texttt {<}$pose$\texttt {>}$, $\texttt {<}$parent$\texttt {>}$, $\texttt {<}$child$\texttt {>}$, $\texttt {<}$axis$\texttt {>}$, $\texttt {<}$axis2$\texttt {>}$, $\texttt {<}$sensor$\texttt {>}$

A $\texttt {<}$model$\texttt {>}$ may have zero or more $\texttt {<}$link$\texttt {>}$ elements, with a mandatory name attribute. A $\texttt {<}$link$\texttt {>}$ may have several child elements such as $\texttt {<}$pose$\texttt {>}$, $\texttt {<}$collision$\texttt {>}$, $\texttt {<}$visual$\texttt {>}$, and $\texttt {<}$sensor$\texttt {>}$, for instance. We describe the SDF tags as needed.

SDF elements may have a local coordinate frame. For those, we can describe their pose (that is, position and orientation) by defining the translation and rotation of their local frame relative to a reference frame. This can be the frame of any other element. We write $\texttt {<}$pose$\texttt {>}$x y z r p w$\texttt {<}$/pose$\texttt {>}$ for a pose definition where x, y, and z specify the translation of the position (in metres) between the local and reference frames, and r, p, and w (roll, pitch, and yaw) are Euler angles (in radians) that define the rotation between those frames. By default, the pose of an element is defined with respect to the frame of its parent. As an exception, the $\texttt {<}$pose$\texttt {>}$ of a $\texttt {<}$joint$\texttt {>}$ is, by default, with respect to the frame of its $\texttt {<}$child$\texttt {>}$ link.

In Fig. 15, the $\texttt {<}$pose$\texttt {>}$ of 1-Wheel defines that its z coordinate is (approximately) 0.06 m above the z coordinate of the $\texttt {<}$pose$\texttt {>}$ of BaseModule. The model $\texttt {<}$pose$\texttt {>}$ definition puts it 0.025 m up with respect to the frame of the world in which BaseModule is used. (This refers to the centre of the wheel, and so ensures that the robot is on the floor, not into it.)

A $\texttt {<}$link$\texttt {>}$ can have zero or more $\texttt {<}$collision$\texttt {>}$ elements, so that it can compose individual shapes with different physical properties to define more complex shapes. The $\texttt {<}$collision$\texttt {>}$ element of a $\texttt {<}$link$\texttt {>}$ has an obligatory name attribute. For example, 1--Wheel is a collision defined in Fig. 15 to describe a cylinder for 1-Wheel.

A $\texttt {<}$collision$\texttt {>}$ has one obligatory $\texttt {<}$geometry$\texttt {>}$ element that describes the shape of the collision object. A $\texttt {<}$geometry$\texttt {>}$ may have optional elements indicated by tags such as $\texttt {<}$box$\texttt {>}$, $\texttt {<}$cylinder$\texttt {>}$, $\texttt {<}$sphere$\texttt {>}$, and $\texttt {<}$mesh$\texttt {>}$. In Fig. 15, the $\texttt {<}$geometry$\texttt {>}$ element of 1--Wheel has a $\texttt {<}$cylinder$\texttt {>}$ whose radius is approximately 0.025 m. A $\texttt {<}$collision$\texttt {>}$ may also have an element $\texttt {<}$laser_retro$\texttt {>}$ for describing the intensity value returned by a laser sensor, and an element $\texttt {<}$surface$\texttt {>}$ for specifying the physical properties of the shape surface, such as friction.

Finally, a $\texttt {<}$link$\texttt {>}$ can have a $\texttt {<}$sensor$\texttt {>}$, which has mandatory name and type attributes. SDF supports several types of sensors. In addition, configuration data of a specific type of sensor is recorded in specific tags such as $\texttt {<}$camera$\texttt {>}$, $\texttt {<}$gps$\texttt {>}$, $\texttt {<}$ray$\texttt {>}$, and $\texttt {<}$sonar$\texttt {>}$ inside the $\texttt {<}$sensor$\texttt {>}$ tags. A $\texttt {<}$sensor$\texttt {>}$ can have its own $\texttt {<}$pose$\texttt {>}$.

A $\texttt {<}$model$\texttt {>}$ may contain $\texttt {<}$joint$\texttt {>}$ elements. SDF supports some types of $\texttt {<}$joint$\texttt {>}$, such as revolute, prismatic, ball, screw, universal, and fixed. In Fig. 15, 6-Hinge is a revolute joint. A $\texttt {<}$joint$\texttt {>}$ names a $\texttt {<}$parent$\texttt {>}$ and a $\texttt {<}$child$\texttt {>}$ $\texttt {<}$link$\texttt {>}$. The $\texttt {<}$joint$\texttt {>}$ is fixed to the parent $\texttt {<}$link$\texttt {>}$, and defines the movement of the child$\texttt {<}$link$\texttt {>}$. Besides a $\texttt {<}$pose$\texttt {>}$ and a $\texttt {<}$sensor$\texttt {>}$, a $\texttt {<}$joint$\texttt {>}$ may have elements based on its type. For example, if a type has one or more degrees of freedom, such as the prismatic or revolute2, we can use $\texttt {<}$axis$\texttt {>}$ and $\texttt {<}$axis2$\texttt {>}$ to specify the unit vector along the axis of rotation and translation.

Next, we compare RoboSim and SDF.

### RoboSim versus SDF

In this section, we in addition indicate a number of ways in which the design of RoboSim improves on that of SDF so that RoboSim p-models are much more concise, readable, reusable, and useful artefacts than SDF documents. The main distinctive features of RoboSim p-models are its diagrammatic nature and the possibility of defining block behaviours. RoboSim is not a front-end for SDF, although it has an associated technique to generate SDF documents automatically to facilitate simulation (see Sect. 5.3). Notably, from a p-model, we can generate automatically a mathematical model that uses the equations and other information in the diagram to enable reasoning (see Sect. 6).

In SDF, containment is captured implicitly: All the elements of a model are contained in the component it implicitly defines. If a nested model is defined, the effect is including its elements in the complete model. There is no requirement for the nested model to represent a self-contained component. It may, for instance, include a disparate set of elements, such as, a camera, a joint, and a nose. An attribute of a link, namely must_be_base_link, can be used to identify a robotic platform, but there is no requirement for the existence of a link with such attribute. Moreover, this attribute does not capture containment, which is a hierarchical relationship. In contrast, RoboSim adds structure to models to improve readability and encourage reuse via novel mechanisms: parts, indexing, parametrisation, referencing, fragments, and library definitions.

SDF elements can define and name a frame of reference. The pose of an element can then be defined in terms of any such frame. In RoboSim, implicit definitions of frames of reference based on the containment relationship minimise the amount of definitions needed, and eases understanding. With the pose as an attribute of the containment relationship, associated with a realisation, rather than a definition, of an element, reuse of definitions in libraries is easier.

Links cannot be connected directly in SDF, but can be connected by fixed joints. RoboSim removes the need for this artifice; three forms of connections (containment, flexible, and fixed) allow clearer and explicit modelling of the relationships between elements of the model.

SDF has support to specify links with properties of microphones, speakers, and batteries. There is no notion of actuator. In contrast, in RoboSim, a library of blocks defines equations for sensors and actuators with such properties [51]. Moreover, new sensors and actuators can be defined by specifying their behaviour equationally.

To summarise, RoboSim is not a graphical front-end for an XML notation, but SDF and other similar notations are very useful. Next, we describe how we support verification by simulation via automatic generation of SDF documents.

### Automatic translation from RoboSim to SDF

Translating a RoboSim p-model to SDF is time-consuming and error-prone if not automated, especially for complex models. In this section, we describe rules that map block types in RoboSim into SDF elements and their attributes. In total, we have 42 model transformation rules given in [51], where we also present a metamodel for SDF. The implementation of these rules (described in Sect. 8) allows automatic translation from RoboSim p-models to SDF documents. This significantly facilitates the automation of testing via simulations by reducing the need for manual intervention for setting up simulations.

The translation addresses several challenges. In general, all the structuring mechanisms used in the p-model are captured by explicit descriptions of corresponding SDF elements. There is no notion of parts in SDF, so the translation of a p-model that uses instances of parts uses the translation of the p-models for those parts. For indexed elements, the SDF definitions are replicated, since again indexation is not part of SDF. Equations are disregarded in the translation to SDF, but annotation templates and their instantiations are considered instead to improve automation. The poses of all elements are calculated and given explicitly with respect to one frame of reference. These include the poses for elements in parts and for indexed elements. Similarly, unique SDF names for all elements are identified.
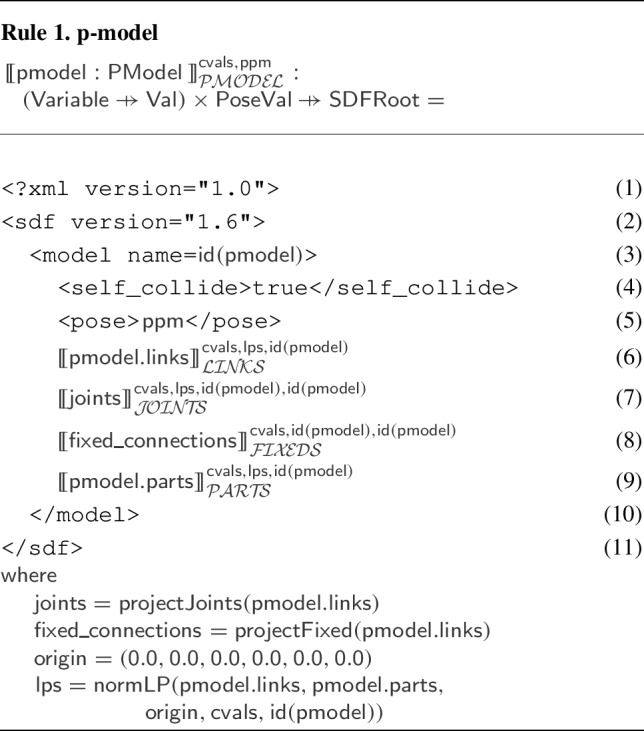


The overall translation is defined by Rule 1. Each rule defines a function, by declaring its name, the name and type of its arguments, its return type, and a body that specifies an SDF term of the return type. In Rule 1, the function defined is . It takes as argument a p-model : an element of the RoboSim metamodel class . Another argument, , is a function mapping uninitialised constants used in  to their values, given as input to a translation. The final argument, , defines a value for the pose (as a tuple of six real numbers) of the p-model in a scenario of interest. The example in Fig. 15 is the result of applying Rule 1 to the p-model in Fig. 2 giving as parameter the pose (0.0,0.0,0.025,0.0,0.0,0.0). In this case, all constants are given a value in the p-model.

The result of  is an SDF document describing a robotic platform, which represents an element of the SDF metamodel class . A (simple) SDF metamodel is omitted here and can be found in [51]. The body of a rule describes a value of the return type using a simple metanotation to indicate conditionals and calls to functions defined by other rules. The terms of the SDF document generated by the rule, that is, resulting from an application of its function, are distinguished by the use of teletype font. Other terms, written in grey, are part of the metanotation.

For clarity, in the body of the rules, we describe the SDF elements using the XML notation adopted in SDF documents. This strengthens their correspondence with their implementations (see Sect. 8), which is via model-to-text transformations. The SDF metamodel, however, used to type the rules, has supported the definition and validation of a compositional set of rules.

In the body of Rule 1, lines (1) and (2) define the first two lines of the SDF documents that we generate from any p-model. They specify the XML and SDF versions used. We use version 1.6 for SDF because it is the latest supported by CoppeliaSim. The approach to translation we describe is, however, of general value and useful even in developing translations for URDF, for instance.

In line (3), the grey font used to write  indicates that this is a term of the metanotation, and so it should be replaced by the unique name of the model. This name is the result of applying the function  to . The function  can be applied to any named element of RoboSim to obtain its unique identifier.

Line (4) defines that the value of the element $$\texttt {<}  \texttt {self\_}$$$$\texttt {collide>}$$ is set to true. By default, SDF assigns a false value to this element. If set to true, all elements in the model can collide with one another except those that are connected by a joint. This is the right behaviour for a physical object.

Line (5) defines a $\texttt {<}$pose$\texttt {>}$ for the model using the parameter . We recall that, in the RoboSim models, the implicit frame of reference of the p-model is given by position (0.0,0.0,0.0) and orientation (0.0,0.0,0.0). Using $\texttt {<}$pose$\texttt {>}$, we position the p-model as required, but keep the implicit frame of reference for the SDF elements.

In Fig. 15, the link 1-Wheel corresponds to the Wheel link in the p-model for LTreel (see Figs. 2 and 7). Its pose results from the composition of the pose of LTreel in BaseModule with that of Wheel in the p-model Treel.

In lines (6) to (9), functions defined in other rules, explained below, are used to determine the translation of the links, joints, fixed connections, and parts of . The joints and fixed connections are those that are contained in the  of , identified by the simple projection functions  and  (omitted here). Flexible connections are translated as part of the translation of the joints and parts that contain them. Fixed connections, however, are encoded in SDF using (extra fixed) joints, and so are translated separately by a function .

The functions applied in lines (6), (7), and (9) have, besides , an extra argument  defined in the  clause of Rule 1. This is a mapping that associates the identifier of each link in , that is, each link directly contained in  or contained in a part of , with its pose, defined with respect to the frame of . For our example,  defines a pose for Core, and for the Wheel and Track links in LTreel and RTreel.

With , we can translate the joints and parts, as well as the links, independently, as defined in Rule 1. The translation of links (line (6)) considers each link directly included in the . Their poses could be calculated independently, but, for uniformity, they are included in . For our example,  gives pose (0.0 0.0 0.057 0.0 0.0 0.0) for $\textsf {Core}$ as already indicated in the p-model.

Similarly, in the translation of joints, as defined in line (7), we consider each joint of the p-model. In this case, however, the pose of each joint needs to be defined in the SDF document with respect to the frame of the link to which the joint is flexibly connected. So, in our example, to translate the joint LHinge, we need the pose of the link Wheel inside LTreel. In line (7), this information is passed on to  in . For our example,  includes the pose (0.0 0.06 0.025 -1.57 -0.0 0.0) for the Wheel inside LTreel. This corresponds to the result of the composition of the poses of the Wheel with respect to Treel and of LTreel with respect to BaseModule, as previously explained.

The translation of a part (line (9)) is in many ways similar to that of a p-model, and so  is also required as an argument of . Translating a fixed connection (line (8)) does not require the argument because the poses of links connected by a joint that does not move do not matter.

Another argument for all functions used in lines (6) to (9) is the identifier  of the ; it is used to determine identifiers for all elements in . For example, these identifiers can be qualified names defined based on the containment relationship. For instance, BaseModule::LTreel::Wheel identifies one of the two Wheel links in BaseModule. In Fig. 15, we have elided the qualification of the names for conciseness, but have used unique identifiers instead.

For joints and flexible connections, the translation requires two identifiers to construct qualified names: the first for the container (p-model or part) of the joints or connections, and the second for the container of the links to which they are connected. In Rule 1, both are , since this is the rule for the platform p-model, which contains all elements. In general, as illustrated later in this section, when translating a part, the container of the links to which the joints or connections of the part are connected may be different.
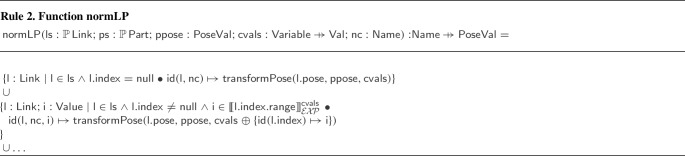

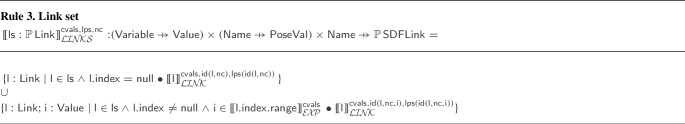


The value of  is the result of a normalisation, captured by the function  defined in Rule 2, applied to all links  and parts  of the . There are three extra arguments to . First, we have the pose of the p-model that contains the links and parts in its two first arguments; in Rule 1, it is , the frame of reference of the p-model also defined in the  clause. Next, we have the value  of the constants, which may be used in defining the poses. Finally, we have the name of the p-model,  in Rule 1, used to uniquely identify the links in the resulting mapping .

In Rule 2, just sketched here, the mapping characterised by  is the union () of four mappings. We show the first two, which consider the links  in the set  given as argument. In the first mapping, we consider the case in which  does not have an index (). So, the mapping associates the identifier  for that link, in the context of the model with identifier , to its pose. We note that  is an overloaded function that may take as an extra argument an identifier for a container element, here , to determine the unique identifier of its first argument. The (standard) function  determines the pose of , via a transformation that takes into account the pose  of the enclosing p-model given as argument. The pose of  is  and may refer to the constants in the argument .

The second mapping is for links  with an index. For each possible value  of the index, we add an element to the mapping that records a pose for an extra link. The possible indices are determined by a function  that evaluates the set  defining the index. The standard definition of expression evaluation captured in  is omitted here. In the mapping, the identifier for the link  considers the value . In the pose calculation, the constant mapping  is enriched (operator ) to record the value  for the index , which can be used in .

The next two similar omitted mappings are for links inside a part  in the argument set .

In Rule 1, line (6), a function  defined in Rule 3 is applied to the set  of  elements. The result is a set of  elements describing links in an SDF document as determined by the function . For each link  in the argument set ,  takes as extra arguments its name and pose. If  is not indexed, as said, its name  identifies  in the p-model or part  where it occurs. If  is indexed, it actually represents a collection of links, one for each value  of the index. The names of the links in this collection are given by . The poses are all identified by . This rule, together with Rule 2 (used to define the value of the argument ), capture the meaning of indexing of links.

In line (7) of Rule 1, a function  is applied to the  in . They are characterised in the  clause by applying the function  to . The joints  identified are those in a   in this set that have a  connection to another link, that is, for which . Because of the well-formedness condition *PMap*1, in a p-model for a robotic platform, the joints that are not connected to a link are inside a part, which has the connection to the link. Such junctions are translated to SDF in the translation of the part. The characterisation of a joint  in  is by a pair including  and its containing link . The definition for  is similar to Rule 3 for links.

In line (8) of Rule 1,  is applied to the  characterised in the  clause by applying  to . The definition of this function is similar to that of  described above. Finally, in line (9) of Rule 1,  is applied to the parts  in . The rules for  and  are similar to Rule 3.

Rule 4 defines ; it characterises an  using the $\texttt {<}$link$\texttt {>}$ tag. The name  of the link given as argument is identified in line (1). In line (2) the pose is defined using the argument ; here, there is a slight abuse of notation, since a  is a tuple, and in SDF, we include a list of numbers. The inertial needs to be calculated; if it is given in the definition of , that is the (straightforward) result. Otherwise, it needs to be calculated from the definition of the bodies of . In the  clause, we apply a function , whose standard definition is omitted here, to  to define a tuple  with three elements , , and  characterising the pose , inertia matrix , and mass  of the inertial component. These are used to define on lines (3)–(14) the pose, inertia, and mass tags for the link’s inertial tag. The (symmetric) inertia matrix is represented by a tuple of six elements.
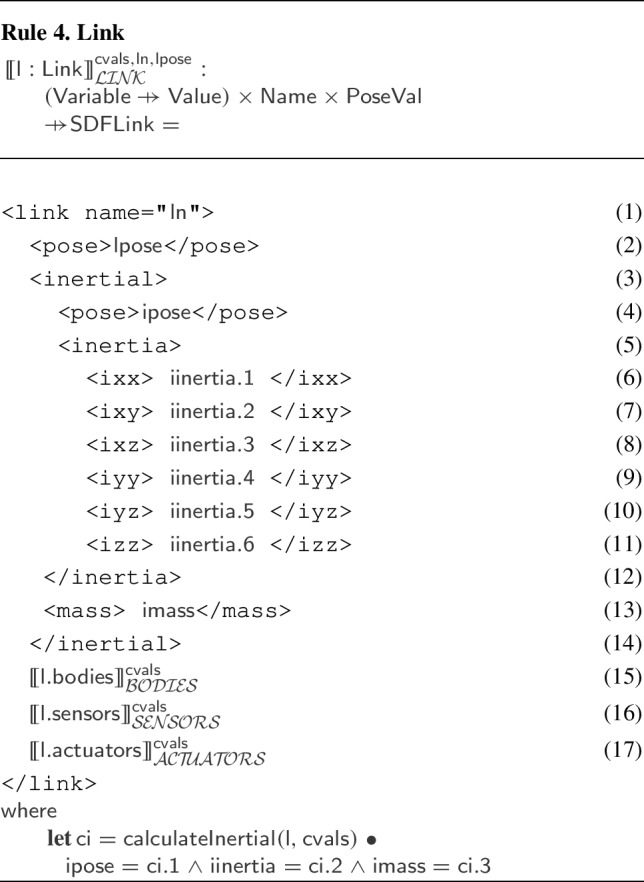


Other elements in link are identified by other functions to specify the link’s bodies (), sensors (), and actuators (). Their definitions are similar to that in Rule 3 for links, applying to individual elements functions , , and . A difference is that, for indexed elements, the argument  is enriched to include the value of the index. This is needed because their poses, whose definitions typically use the index, still need to be calculated. For bodies, sensors, and actuators, there is no table like  in Rule 4. (We use  to simplify translation of joints, whose poses in SDF are defined with respect to the frame of reference of the link to which it is flexibly connected, not of the link that contains it. So, the translation of a joint, as explained, cannot be part of the translation of the link that contains it.)

The translation rules for a joint, a sensor and an actuator capture the meaning of annotations. We present here Rule 5 for a joint. The parameters of the function  include, besides the mappings  and , the link  that contains , the names  and  of the containers of  and of the link to which  is flexibly connected, and the flexible connection  for . We recall that  may be contained in  or in a part containing .
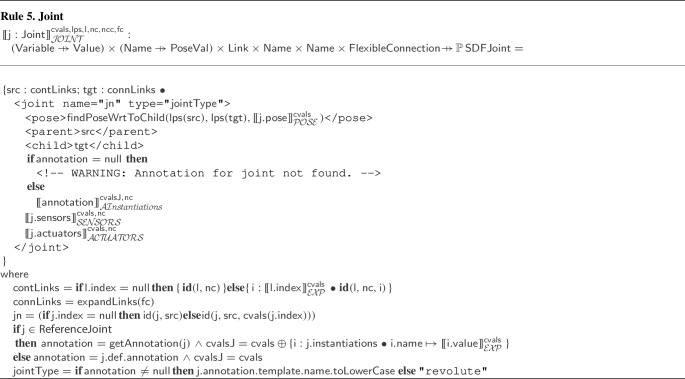


Applying Rule 5 results in a set of SDF joint definitions (that is, elements of ) because, if the link  in which  is contained is indexed, or if the flexible connection  for  is contained in an indexed part or connected to an indexed link, then  actually defines a collection of joints. As defined in Rule 5, this set contains a joint for each pair (, ) taken from the sets , of names of container links defined by , and , of names of connected links defined by .

The set  is defined in the  clause of Rule 5. It contains just  if  is not indexed (). If  is indexed, then we have a name  for each value  of the index. The set  is also defined in the  clause using a function  omitted here. It caters for indexation in the part containing , if any, and in the link to which  is connected, defining a set of link names, as appropriate.

The joint definition in the body of Rule 5 gives its name  and its type . The value of , defined in the  clause, is just the name of  as defined by , if  is not indexed. Otherwise,  is applied to an extra argument : the value of the index of  recorded in . We recall that the function  for sets of joints (see similar Rule 3 for links) uses  to record a value for the index in the translation of each instance of an indexed joint.

Since SDF does not have a general mechanism to define the equations of joints, like RoboSim, we require an  to define all but revolute joints. The  clause defines the . If there is an  for the joint (), its  is given by the name of the annotation template (see Fig. 4). (So, RoboSim p-models are not tailored to SDF, but we can use the annotation library of templates to customise translation for SDF. Moreover, we can define libraries for other XML-based notations.) If the joint has no , we define the  to be revolute by default.
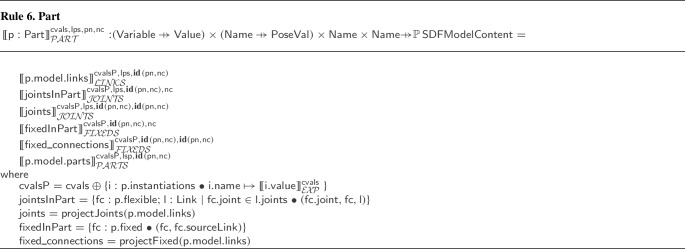


If  is a reference to a joint definition (that is, ), rather than a inition itself, then the annotation can be in the definition or in the reference. The annotation in the reference, if present, overwrites that in the definition, if any. In the  clause, the function  is used to define  taking this into account. If,  is not a reference, the  is that in the inition of .

If  is a reference, it may contain instantiations  of constants, which are used to define a new mapping  of constants. This mapping enriches  by mapping each constant  in an instantiation  to its value .

The tags parent and child identify the container link  and the connected link . The pose of the joint is defined with respect to the child link, as required in SDF. The pose transformation is achieved using the (omitted) function , which takes the poses of the containing and connected links  and , and the pose of . (We recall that  is originally defined in RoboSim with respect to , but needs to be defined with respect to  in SDF.)

If there is no , we include a comment (indicated by $\texttt {<}$!--... --$\texttt {>}$) in the SDF document to flag that. The SDF document generated is valid, and the points where perhaps it needs to be extended are indicated explicitly. Ideally, however, the extension should be done via annotations, not by modifying the automatically generated document. If there is an annotation, the translation of the  leads to the introduction of tags that reflect the default values in its template and its instantiations. This is handled by the function . For example, in Fig. 15, the element defined by the tag axis and all the child elements of axis are defined by translation of an annotation (omitted in Fig. 2).

Finally, in Rule 5, the translation of any sensors or actuators included in the joint is defined using functions  and .

The translation of a sensor also uses annotations. Like for a joint, if a sensor  has no annotation, a warning is produced. Otherwise a $\texttt {<}$sensor$\texttt {>}$ is defined: its name  and pose  are those of . The  is defined by the name  of the template.

The translation of actuators is simpler. There is no general tag for actuators in SDF. The type of the annotation template defines a name for a tag.

The translation of a part, defined by Rule 6, is similar to that of a p-model. It is the result of the translation of the links, joint, and fixed connections of the part, as well as of any additional parts used to define the part itself.

Applying Rule 6, we get a set of definitions of links, joint, sensors, and actuators. (These are collectively represented by the class  in the SDF metamodel.)

The constant mappings  used in the translation of the part elements is defined in the  clause of Rule 6. It extends  by adding values  for each constant  included in the instantiations of the part  being translated.

The translation of the joints and fixed connections of is defined by  and , like in Rule 1. These functions, however, are applied to two disjoint sets of joints and fixed connections. In the sets  and  (defined in the  clause), we have those contained in the links of the  for . Like in Rule 1, they are determined by projection of the components of the links of the part .

In contrast, the sets  and  (also defined in the  clause) have those that are associated with a (flexible or fixed) connection  for the part , rather with a link of . When translating these sets, the identifiers for the links to which they are connected, passed as arguments to  and , are different. They are the identifiers  of the part’s container, rather than that of the part itself.

**Fig. 16:**
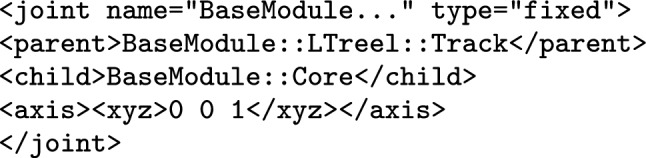
Example of SDF encoding of a fixed connection in Fig. 2: that between LTreel and Core

For instance, in our example in Fig. 2, we have a fixed connection that belongs to the part LTreel (and similarly for RTreel). In its translation, we use arguments BaseModule::LTreel and BaseModule. The former is the container of the connection, that is, the part, and the latter is the container of the link to which it is connected, that is, Core. The result of the translation is the SDF definition of a joint partially shown in Fig. 16.

We recall that a fixed connection is encoded in SDF using a joint of type fixed. Its unique qualified name (elided in the example in Fig. 16) is formed from the qualified names of the links that are connected. The link in the container part becomes the parent link of the joint in SDF. In defining its qualified name, we use the name of the part: the first name argument given to  in Rule 6. The link to which the connection is connected becomes the child link. To define its qualified name, we need to use the second name argument of . The definition of the axis is a requirement of SDF, but has no consequence for a fixed joint. The translation uses an arbitrary value 0 0 1.

In Sect. 8, we discuss the implementation of all the rules in RoboTool. First, we consider a different kind of artefact that can be generated from a p-model: a mathematical description.

## Mapping to hybrid state-rich CSP

**Fig. 17:**
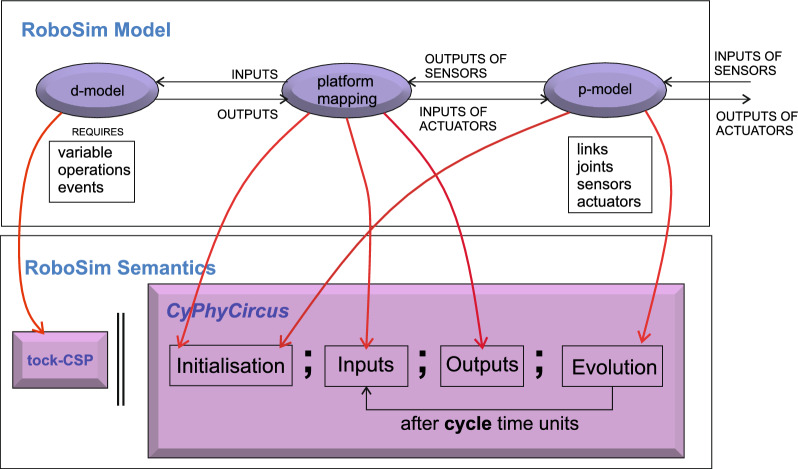
Structure of the semantics of a complete RoboSim model

Automatic generation of SDF documents supports analysis via simulation. Here, to complement testing via simulation, we present another model transformation technique to generate *CyPhyCircus* models for proof of properties of the robotic system that takes into account the platform.

As previously said, a p-model and a platform mapping are components of a RoboSim model, which also includes a d-model. A semantics for a complete RoboSim model needs, therefore, to capture the meaning of all these components. Nevertheless, the semantics is compositional, and the different components can be used independently of each other. The semantics of d-models [19] is already described in a discrete time variant of CSP, namely tock-CSP [5, 62]. Here, due to the continuous nature of the behaviour of physical systems, we need to account for both discrete and continuous behaviour. So, we use a hybrid version of CSP, called *CyPhyCircus* [30, 53], to describe the semantics of p-models and platform mappings. The composition of the d-model’s CSP semantics and the p-model’s *CyPhyCircus* semantics is justified via the Unifying Theories of Programming (UTP) [36]. *CyPhyCircus* is based on the works in [38, 44], but deals with variables and has support for automated proof in Isabelle [55].

In Sect. 6.1, we describe the structure of the semantics of a RoboSim model. In Sect. 6.2, we detail the semantics of a p-model and its platform mapping via model transformation rules.

### Overview of RoboSim semantics

In CSP, mathematical models are processes. These are mechanisms that capture allowed and required patterns of interaction. In the case of a *CyPhyCircus* process, it also captures the trajectories of real-valued state variables. With *CyPhyCircus* processes, we can model the reactive and continuous behaviour of a mechanism. Like in CSP, reactions (or interactions) are captured by atomic and instantaneous events. Processes communicate with each other using such events.

The semantics of a complete RoboSim model has the structure of processes shown at the bottom in Fig. 17. There are two processes composed in parallel ($\parallel$). The first is the tock-CSP process that encodes the semantics of the d-model [19], and the second is a *CyPhyCircus* process that specifies the behaviour of the p-model and its interaction with the process for the d-model, also reflecting the definitions in the platform mapping.

The hybrid p-model process acts on a set of variables over time, which, in general, correspond to physical quantities, such as distance and torque, used as inputs to sensors, and outputs of actuators and joints. It also uses events to communicate with the d-model process. Its definition is via a combination of *CyPhyCircus* actions in sequence (;) as depicted in Fig. 17. Action is the *CyPhyCircus* name for a state-rich CSP-like process that can, however, use and update variables, and is local to a *CyPhyCircus* process, here the p-model process.

A p-model process first defines the initial values of its state variables (Initialisation), and then proceeds iteratively, with each iteration capturing the behaviour of one simulation cycle. An iteration sends updates to the d-model process via its input events (Inputs); reads requests from that process via its output events, variables, and operations, and resets a timer variable (Outputs); and finally evolves the system of differential equations for the duration of the cycle (Evolution).

**Fig. 18:**
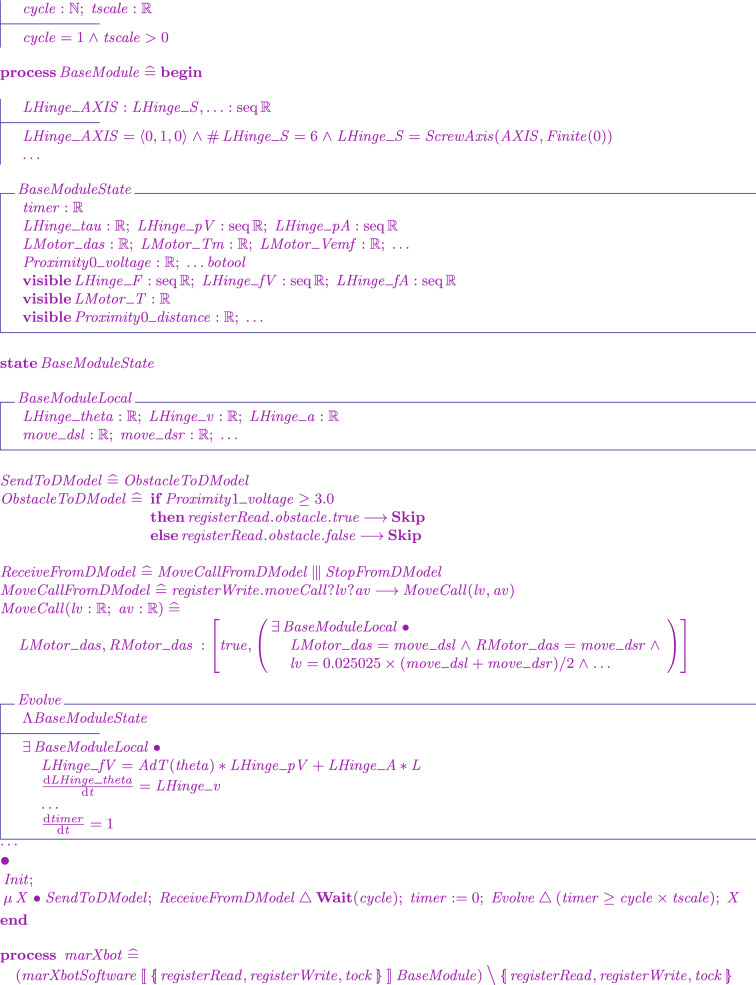
Sketch of the *CyPhyCircus* model for the marXbot (color figure online)

For the RoboSim model in Figs. 1, 2, and 8, the semantics is shown in Fig. 18 at the bottom. As indicated in Fig. 17, this is a process, called *marXbot* here, that composes in parallel (indicated by the symbols  and ) the d-model process *marXbotSoftware*, which gives the semantics of the RoboSim d-model (Fig. 1), and the p-model process *BaseModule*, which actually specifies the semantics of the p-model itself and its associated platform mapping (Figs. 2 and 8).

We omit the definition of the tock-CSP process $marXbotSoftware$, since its details are not relevant to understand the semantics of a p-model and a platform mapping. It suffices to know that d-model processes use events *registerRead* and *registerWrite* to read inputs and communicate outputs (including operation calls), and an event *tock* to represent the passage of time according to the software’s discrete clock. So, these events are also used by the p-model processes.

In the parallelism that defines a complete system, such as that defining the process *marXbot*, the parallel d-model and p-model processes communicate (synchronously) internally. The visible behaviour is the passage of continuous time, and the evolution of the inputs to the sensors, and outputs of the actuators and joints. The parallelism definition establishes that the processes communicate on the events *registerRead*, *registerWrite*, and *tock* (indicated between the symbols  and ). Their repeated mention after the symbol  indicates that such communications are internal (hidden), that is, interactions with the parallel process as a whole cannot be via these events. They are just for communication between (the mathematical models of) the control software (d-model) and the robotic platform (p-model).

The process *BaseModule* for our example is sketched in Fig. 18; we describe its definition in detail. Instead of being defined in terms of other processes, a p-model process is defined by declaring, between ${\textbf {begin}}$ and ${\textbf {end}}$ keywords, a state and a main action that defines its behaviour. The state is declared in a clause ${\textbf {state}}$. The main action comes at the end, between a $\bullet$ and the ${\textbf {end}}$.

The ${\textbf {state}}$ clause names a *CyPhyCircus* block, called schema, like the similar construction of the Z notation [67]. The state schema is defined separately to declare the private and visible state variables of the process. In our example, it is named *BaseModuleState*. In that schema, the private variables include a *timer* variable that records the time elapsed in each iteration, the inputs of actuators and joints, and the outputs of sensors. These variables are private to the p-model process (see Fig. 17), so their values are not visible to other processes. For example, inputs of actuators are for communication from the platform mapping to the p-model. In Fig. 18, we show, for instance, the variables for the inputs (tau, pV, and pA) of the Revolute joint LHinge (see Fig. 4).

The state schema also declares the visible variables. These represent the values of the inputs of the sensors, and the outputs of the actuators and joints. Their declaration uses the ${\textbf {visible}}$ modifier. In Fig. 18, the definition of *BaseModuleState* shows the declaration of some of these variables. We show, for example, the declaration of the variable $LMotor\_T$ for the output of the LMotor, and of the variable $Proximity0\_distance$ representing the input to the sensor of index 0.

The types of the variables in the state of a p-model process are defined using standard types like $\mathbb {R}$ (real numbers) or sequences of $\mathbb {R}$ ($\text {seq}\,\mathbb {R}$) to represent vectors and matrices. They are, however, functions over time. A variable *v* of type *T* corresponds to a function of type .

The local variables declared in all blocks of the p-model and platform mapping are also declared in the p-model process, together in a separate schema: *BaseModuleLocal* in the example. In Fig. 18, we show the variables to represent local variables of LHinge (theta, v, and a) and of the move block of the platform mapping.

The constants declared in all blocks (of both the p-model and platform mapping) are defined locally in the process as well. In Fig. 18, at the top of the definition of the process *BaseModule*, we show the declaration of a few constants for LHinge. These definitions reflect the library definition for Revolute and the instantiation of its constants in the realisation LHinge, which defines the value of AXIS. They use a *CyPhyCircus* library of definitions in which functions like *ScrewAxis* are specified via pre and postconditions.

It is the trajectories of the values of the ${\textbf {visible}}$ variables that are specified by the p-model process, as well as the communications with the d-model process via the channels *registerRead* and *registerWrite*. That specification is given by the main action in terms of local actions *Init* (corresponding to Initialisation in Fig. 17), *SendToDModel* (Inputs in Fig. 17), *ReceiveFromDModel* (to define Outputs), and *Evolve*, all defined in the body of the p-model process ($BaseModule$ in the example).

We omit the simple action *Init*: a sequence of assignments that define the (initial) values of the private state variables. In the main action, after *Init*, we have a recursion defined by the operator $\mu X \bullet A;X$ that declares an action *A*, gives it the local name *X*, and calls *A* recursively once *A* is finished. In the main action of a p-model process, *A* defines the behaviour of a cycle of simulation. At each step of the recursion, that action sends input events to the d-model process using the action *SendToDModel* and then reads requests from the d-model using the action *ReceiveFromDModel*.

When the d-model tock-CSP process completes its computation for the current cycle, one or more *tock* events signal that the software (d-model) simulation clock is advancing to the next cycle. So, in the main action of the p-model process, *ReceiveFromDModel* is interrupted () by a ${\textbf {Wait}}(cycle)$ action. The global constant *cycle* is a positive natural number that defines the cycle of the d-model in terms of a number of time units, that is, *tock* events. If the d-model defines or constrains the value of the cycle, the global constant *cycle* records that value or constraint. In our example, *cycle* is 1, as defined in Fig. 1. The action ${\textbf {Wait}}(cycle)$ monitors and accepts *cycle* *tock* events, taking over when the first *tock* occurs and finishing after the last *tock*.

Following ${\textbf {Wait}}(cycle)$, the main action of a p-model process resets its own *timer*, and evolves the system of equations using an action *Evolve* to define the trajectories of the visible variables.

In accordance with the simulation paradigm, the actions *SendToDModel*, *ReceiveFromDModel*, and ${\textbf {Wait}}(cycle)$ take no simulation time. The software clock, captured by *tock* events, is internal and does not interfere with the continuous simulation time defined by the p-model process. Time passes, however, during *Evolve*. Another interrupt terminates *Evolve* as soon as the *timer* expires, that is, its value exceeds $cycle \times tscale$. Here, another global constant *tscale* defines the correspondence between a discrete time unit and the continuous time. For instance, if the value of *cycle* is 5 (time units), and *tscale* is 0.5 ms, then the actual cycle of the simulation is 2.5 ms.

The value of *tscale* is not defined in a p-model, and can be left open as in our example, or provided as input to the automatic generation of the semantics and fixed. The use of the notion of time units allows model independence with respect to both simulation and deployment time.

The semantics of the platform mapping is captured by the actions *SendToDModel* and *ReceiveFromDModel*. *SendToDModel* communicates to the d-model process information about each of the inputs through the *CyPhyCircus* *registerRead* events. *SendToDModel* determines whether a RoboSim input event has occurred, and outputs that information via *registerRead* as a boolean. If the input communicates a value, it is carried by *registerRead*, too. If there are several inputs, the information can be sent in any order required by the d-model. In this case, *SendToDModel* is an interleaving of actions that model each of the mappings for input events.

In our example, the single input event of the d-model is obstacle. So, the definition of *SendToDModel* is just *ObstacleToDModel*, and there is no need for an interleaving. The conditional that defines *ObstacleToDModel* is based on the definition of obstacle in the platform mapping: see Fig. 8. Depending on the value $Proximity1\_voltage$ of the output of the sensor of index 1, *true* or *false* is communicated by *registerRead*, along with the name *obstacle* of the event. Afterwards, the action terminates (${\textbf {Skip}}$).

The action *ReceiveFromDModel* is similar. It accepts, in any order, outputs via *registerWrite* events. The platform mapping defines their effect. In our example, we have two operations (move and stop), each defined by an action *MoveFromDModel* and *StopFromDModel* (the latter omitted in Fig. 18). The actions are interleaved ($|||$) to define *ReceiveFromDModel*.

An output action is triggered by the occurrence of a *registerWrite* event communicating the name of the event, variable, or operation for the mapping it models. In the example, *MoveFromDModel* is triggered by an event *registerWrite*.*move*, carrying also the values *lv* and *av* given as argument in the call to move. Its effect is defined by a data operation, in the example *MoveCall*. That action takes any arguments as parameters, and, in the case of a mapping specified by equations, is defined by a specification statement capturing the data update entailed by the equations.

A specification statement first lists the variables that are updated. In our example, these are the inputs to the motors $LMotor\_das$ and $RMotor\_das$. Next, the specification statement gives a precondition for the data operation. In the semantics of a mapping, it is always specified as *true*. Finally, the postcondition is given by the set of equations, where the local variables are quantified. The quantification uses the schema declaring all local variables, but, of course, only those actually used in the mapping are relevant. In the example, these are $move\_dsl$ and $move\_dsr$.

The action *Evolve* is defined by a Z-like schema that specifies how the state is modified. The $\Lambda$ declaration, in our example, $\Lambda BaseModuleState$, indicates that this action modifies the state as specified by a system of differential equations. In our semantics, the equations are taken from the p-model blocks, but the local variables are existentially quantified. This definition differs from the specification statement defining *MoveCall*, for instance, in that it defines trajectories for the state variables, rather than a value update.

As already said, the semantics of a RoboSim model can be calculated by RoboTool. With this model, we can prove properties specified in terms of the inputs of the sensors, and outputs of the actuators and joints. As expected, the behaviour of the embedded software is only perceived through the behaviour of the platform.

For example, the property indicated at the end of Sect. 3 can be precisely described as follows.

Once $Proximity1\_distance$ is less than a value *d*, then, after *t* time units, the values of $LMotor\_T$ and $RMotor\_T$ are 0.

Now, we can refer to elements of the p-model: variables that represent the input $Proximity1\_distance$ to a sensor, and the outputs (torques) $LMotor\_T$ and $RMotor\_T$ of two actuators as described above.

In Sect. 8, we describe how we can prove such properties using RoboTool and theorem provers.

### Semantic rules

Like for the transformation to SDF, we have also defined rules that specify a p-model *CyPhyCircus* process. Such a process is only well-defined for a complete RoboSim model, including not only the p-model block diagram, but also its associated platform mapping diagram. For this reason, Rule 1, which specifies the overall process, defines the function , which actually applies to a PlatformMapping , not just a PModel.
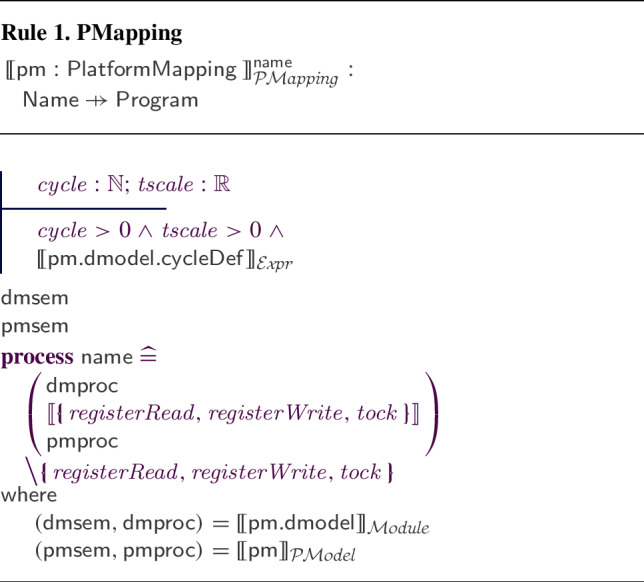

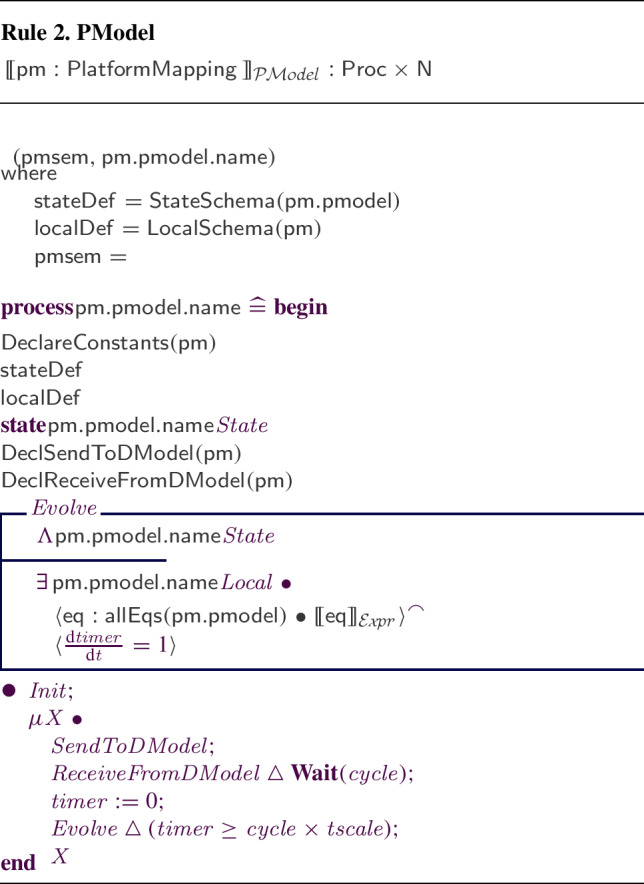


The metanotation used to define the rules here is similar to that used for the rules in Sect. 5.3. The result of the rules in this section, however, are elements of the abstract syntax of *CyPhyCircus*. Moreover, the target notation is distinguished not in teletype font, but in italics as usually adopted for the rendering of *CyPhyCircus* models.

In Rule 1, the result is a *CyPhyCircus* program. It comprises (1) the declaration of the constants *cycle* and *tscale*; (2) the definition of the processes  and  that capture the semantics of the d-model () and of the p-model () in ; and (3) the definition of the overall process that puts them in parallel. The declaration of *cycle* and *tscale* includes, besides the restrictions that their values are greater than 0, restrictions on the cycle potentially included in the d-model, defined by the element . This is a Boolean expression, which is evaluated by the standard semantic function  for expressions. Its definition is that for RoboSim d-models, extended do deal with derivatives and integrals, and special variables like t and myPose, used to denote time and pose of the element represented by the block. In our example, the restriction is that $cycle = 1$. (In Fig. 18, for clarity, we have simplified the restriction $cycle > 0 \wedge cycle = 1$ to $cycle = 1$.)

The semantics of  is given by the function  defined in [19]. The semantics of  is given by Rule 2. In both cases, as indicated in the  clause, we get a pair containing a process and its name. We use the names  and  in the definition of parallelism. The  of that overall process combining the semantics of the d-model of the p-model is an extra parameter of . To generate the process in Fig. 18, we apply Rule 1 to the mapping in Fig. 8 with argument *marXbot*.

In the definition of Rule 2, the result is a pair  containing a p-model *CyPhyCircus* process , defined in the  clause, and its name , which is the name of the p-model itself. To fully define , we use a number of functions defined by other rules in [51]. In the  clause, functions  and  define the schemas that declare the state variables of the p-model process and the variables representing local variables in the p-model and platform mapping elements. In our example, , applied to BaseModule, defines the schema *BaseModuleState* in Fig. 18. *BaseModuleLocal* is defined by  applied to the mapping in Fig. 8.

The process  has the structure already illustrated in Fig. 18. In Rule 2, its constants declaration, and its actions *SendToDModel* and *ReceiveFromDModel* are defined by functions , , defined below, and  in [19]. In the definition of the ${\textbf {state}}$ clause and in the schema *Evolve*, the names of the state and local schemas are taken to be based on the name  of the p-model. In the body of *Evolve*, the equations are characterised by a sequence, delimited by  and . Its last element is the differential equation that specifies the evolution of the variable *timer*. It is concatenated () to the sequence of elements formed from the equations  taken from the sequence  of all equations of the p-model. Each equation is translated by the semantic function  for expressions.

In Rule 3, we give the specification of the function  for a PlatformMapping . Here, we use in the metanotation an indexed interleaving operator (). It denotes the actual interleaving of the actions  from the set  defined in the  clause. In this set, we have actions  that give semantics to the input event mappings  from the set  in . In our example, we recall, there is no interleaving, because the set  is a singleton. In general, like in our example, we expect that there is at least one input. If, however, there are no inputs, the set  is empty, and the interleaving degenerates to ${\textbf {Skip}}$, the action that terminates immediately.
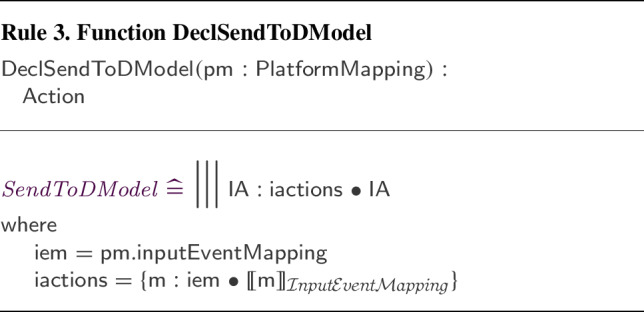


The function  is specified by Rule 4. It defines a *CyPhyCircus* action named after the event in the mapping: its name is given by . The action is a conditional on the Boolean expression defined in the predicate  of the mapping. Two additional functions  and  define the $\,{\textbf {then}}\,$ and ${\textbf {else}}$ branches of the conditional. In our example, the action *ObstacleToDModel* is defined by Rule 4.
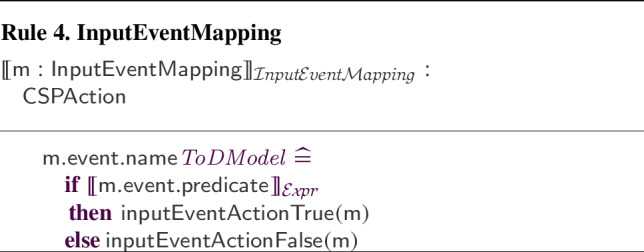


The function  is defined in Rule 5 for a mapping . It considers two cases. In the  clause,  is defined as the parameter of . If the event for  is not parametrised, that is, it does not communicate a value (), then the action defined just communicates the  of the event and *true*. The  is also defined in the  clause. In our example in Fig. 18, the semantics of the mapping for obstacle in *ObstacleToDModel* is covered by this case.

If the event is parametrised, the action is an external choice () over values represented by a variable  named after the parameter. The values are taken from a set defined by . Parametrised events are defined using, besides a predicate that determines when the events occur, a set of equations that defines the values that it communicates. The function  defines these values. If there are several, the external choice communicates one value chosen arbitrarily (because the *registerRead* events are ultimately hidden as shown in Rule 1). Typically, we expect that there is a single value in the set , so that the choice is actually degenerate and only that single value can be communicated via the *registerRead* event. Our semantics, however, makes no such assumption. In addition, if the equations define no values, the semantics introduces a deadlock. So, a deadlock check of the p-model process can identify such problems.
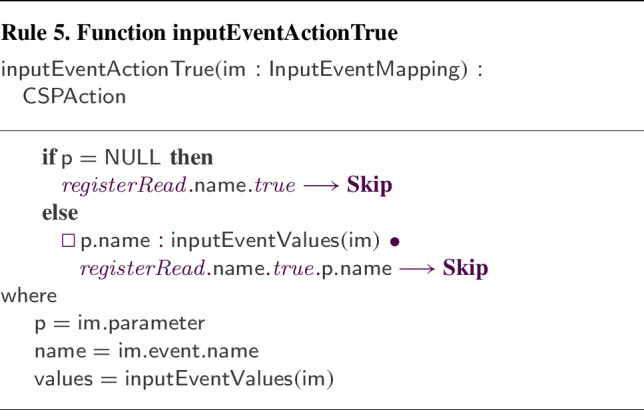


As mentioned, the omitted rules are available at [51]. In the next section, we discuss the implementation of our transformation rules.

**Fig. 19:**
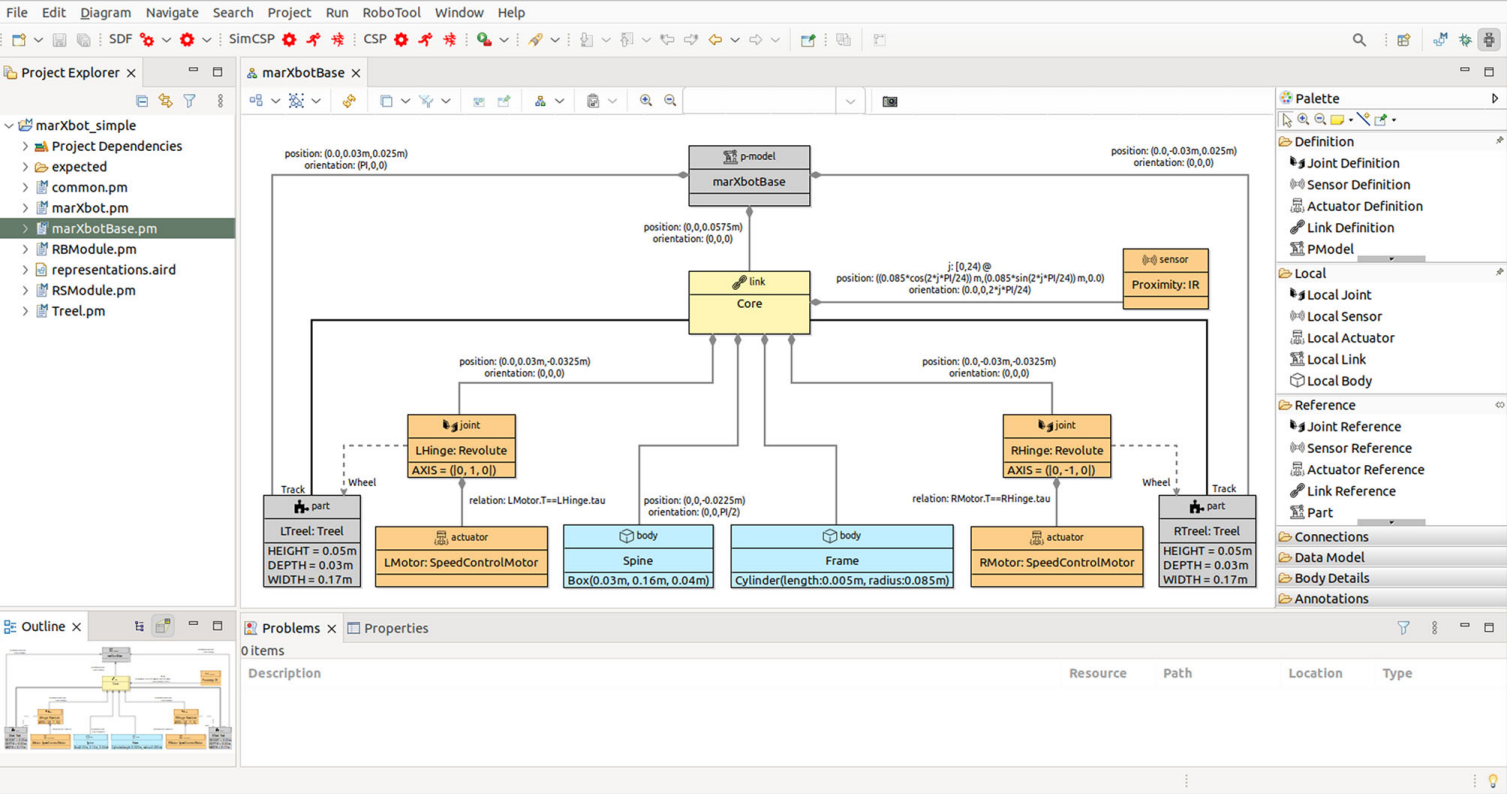
RoboTool graphical editor

## RoboTool

In this section, we present our work extending RoboTool to implement the RoboSim p-models notation: metamodel and well-formedness conditions for edition and validation, the translation to SDF, and the calculation of the semantics. Section 7.1 describes RoboTool and the extensions that support modelling. Section 7.2 describes the implementation of the SDF translation rules. Finally, Sect. 7.3 discusses the implementation of the *CyPhyCircus* semantics of p-models.

### Modelling and Validation

RoboTool^14^ is a set of Eclipse^15^ plug-ins implemented using Xtext^16^ and Sirius.^17^ We have used EMF to implement the metamodel from Sect. 4 to generate a textual editor using Xtext and a graphical editor using Sirius. The textual notation is used as an internal representation. A different implementation of the same metamodel might choose a different representation.

Figure 19 shows a snapshot of RoboTool with the block diagram from Fig. 2 open. The RoboTool window has four areas: model explorer (top-left), outline (bottom-left), graphical editor (top-right), and properties/problems (bottom-right). The graphical editor supports two types of block diagrams: p-models and platform mappings, as described in Sect. 4.2.

The area for the graphical editor itself is divided into two parts: the diagram canvas (left) and the tool palette (right). Block diagrams are constructed in the diagram canvas using tools from the palette; additionally, diagrams can be edited by double-clicking (for example, to edit names and poses), or by right-clicking elements and selecting actions from the menu. The well-formedness conditions described in Sect. 4.3 and type compatibility in expressions and statements are automatically verified.

**Fig. 20:**
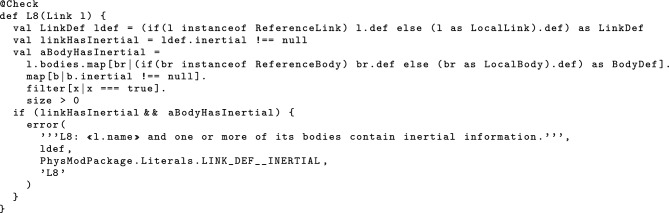
Implementation of the well-formedness condition *L*8 for links

The checking of the well-formedness conditions is implemented using the Xtext validation mechanism. Each condition is associated with one or more validation rules implemented by a method written in Java or Xtend^18^ and annotated with @Check. Figure 20 shows the implementation of the condition L8 for links (see Sect. 4.3).

L8 first identifies the LinkDefinition ldef of its Link argument l covering the cases in which it is a ReferenceLink or a LocalLink. Next, the Boolean linkHasInertial records whether l includes an inertial (ldef.inertial != null). The condition aBodyHasInertial records whether any of the bodies of l defines an inertial. For that, first, with l.bodies we get the collection of Body elements of l. The map method takes a lambda expression and returns a new collection formed from the results of applying that lambda expression to each object of the original collection. In the example, the first use of map gives the collection of BodyDefinitions for the elements br in l.bodies. The second call to map gives a collection of Boolean values b.inertial != null, for each BodyDefinition b. With filter, we keep just the occurrences of true. Finally, aBodyHasInertial, records whether this collection has at least one element (size> 0). Afterwards, L8 checks whether both linkHasInertial and aBodyHasInertial are true, and if they are, it produces an error using the method error. This method takes a textual description of the error, a literal that indicates the element of the abstract syntax tree to which the error should be attached (in this case, ldef) and an identifier for the type of error.

The close correspondence between the well-formedness conditions and the rules implemented in Xtext provides validation for both the language and our conditions. Violation of such rules not only produces error information, but also prevents the generation of simulations and *CyPhyCircus* models. Adding or removing validation rules to cater, for instance, for different semantic models or applications (like code generation) is simple.

### Simulation

**Fig. 21:**
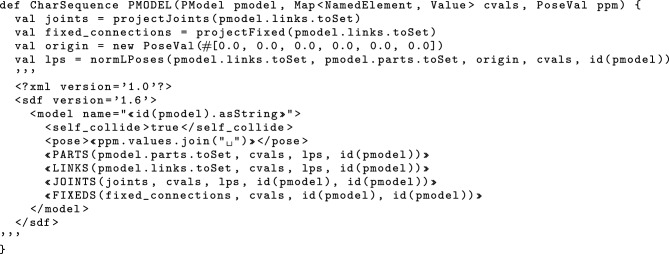
Implementation of p-model to SDF transformation Rule 1

RoboTool implements the model transformation rules of Sect. 5 as Xtend methods to calculate an SDF document for a p-model block diagram. As an example, Fig. 21 shows the Xtend implementation of Rule 1 from Sect. 5.3.

The implementations of the rules are essentially Xtend renderings of their definitions in Sect. 5.3, but we describe a few superficial differences. First, the rules are implemented as model-to-text transformations. So, they all return a string: CharSequence, rather than an element of the SDF metamodel. In each rule, the  clause is implemented via local variables defined at the start of the implementation. The metanotation is Xtend, but given its simplicity, the differences are minor. The target language constructs are encoded in Xtend as strings enclosed in triple quotes, with in-line meta-expressions identified by the use of guillemets («»). Finally, there are some small variations regarding types. The function type is Map in Xtend. Moreover, methods like toSet and asString ensure type compatibility, as we handle collections as sets in our formal rules, and use strings to implement identifiers.

In summary, the implementation in Xtend validates our technique to generate SDF documents, because it matches the definitions of the rules and functions closely. Also, RoboTool has enabled the construction of several examples, and their validation via the use of CoppeliaSim (and Gazebo) to depict the platform defined by the SDF documents automatically generated. The consideration of these examples by hand is virtually infeasible given the size of the SDF documents defined.

**Fig. 22:**
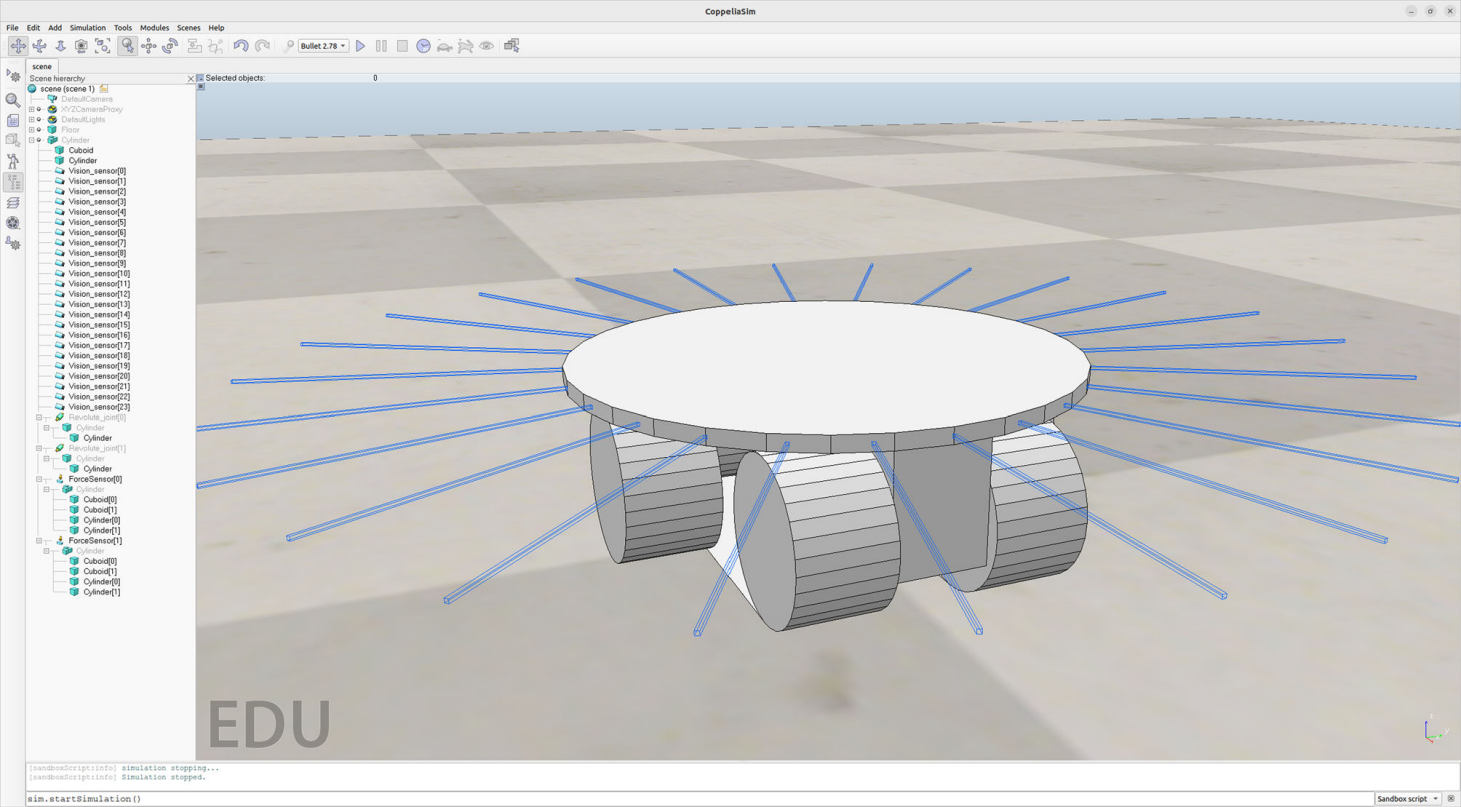
CoppeliaSim—loading and using our automatically generated SDF document

Figure 22 presents the result of loading the SDF document generated for our running example (BaseModule in Fig. 2) in CoppeliaSim. The main panel shows the rendering presented in Fig. 3, but with blue lines indicating the direction of each of the sensors. On the left, a scene hierarchy shows the elements in the scene: a camera, some lights, the floor, and other elements reflecting CoppeliaSim’s interpretation of our SDF document. The rendering shows what the camera captures, given its position and the light. At the bottom, a command line accepts code written in Lua [37]^19^; we can, for example, inspect the elements of the scene, including those defined in SDF. In Fig. 22, the command starts a simulation using the scene. Above, a log gives information about the code execution. In Fig. 22, the log informs that the simulation has terminated.

Since, as already mentioned, a p-model may be defined in terms of constants whose values are not given, the SDF generator asks for a value for these constants. This means we can easily generate multiple SDF documents by varying the values of constants, and it is particularly useful for the use of the simulation in the context of evolutionary robotics and design-space exploration.

Some aspects of the implementation of the SDF document generator needed to be tailored to CoppeliaSim. (So, we envisage that other very similar generators may become useful to deal with different simulators.) For example, qualified names can become long, and CoppeliaSim imposes a limit on the size of element names. RoboTool creates coded short and still unique names for the elements, and produces, alongside the SDF document, a table that maps the qualified names to the coded names (and vice versa).

In addition, we have used SDF 1.6, the latest version supported by CoppeliaSim. The more recent SDF 1.7 comes with semantics for the frame element, and supports custom elements and attributes. As already said, RoboSim, for clarity, fixes the frame of reference for poses using the containment relationship, and our translation does not make use of ad hoc frame elements. So, this change has no impact on our translation. The possibility to define custom elements and attributes also has no impact, but this facility may make RoboSim annotations even more useful, since they are not tied to any SDF element.

### Verification

The *CyPhyCircus* generator is also a faithful implementation of our rules described in Sect. 6.2 as Xtend methods. The output for a well-formed p-model is a model file in the folder cpc-gen. The models produced can be used without extra information being provided by the user. For instance, our p-models allow uninitialised constants in a p-model connected to a platform mapping, allowing the effective modelling of a family of p-models, which can then be analysed to identify, for instance, restrictions over these constants that guarantee a particular property holds.

To facilitate proof, there are several tools that can be used for automatic simplification and solution of equations. Maxima [69] and Maple [1], for instance, are popular tools for symbolic solution. We have enriched RoboTool with a facility for symbolic reasoning developed using the SymPy Python library, which allows us to integrate the assembly and solution of equations alongside the parsing of the symbolic p-models.

This tight coupling between the modelling tools and solvers enables more complete solutions to kinematic equations by allowing model information to be available for more informed equation parsing, simplification, and solution. SymPy is very mature, is used in the SfePy and SageMath scientific libraries, and can handle complex calculus expressions, Taylor series expansions, and general higher-order linear ordinary differential equations with constant coefficients [39].

For each p-model element containing an equation, its input and output variables, and its local variables and constants, are hierarchically extracted from the native XML p-model (representing the metamodel in Sect. 4) into the Python workspace. Variables used in equations are not restricted to a pre-defined set of variables and internal meanings. This allows the solver to handle physical relationships that are abstract and different from conventional robot kinematics. We can also obtain solutions for first- and second-order ordinary differential equations of the most well-known ODE forms used in kinematics.

In the next section, we describe a few example of use of p-models developed using RoboTool.

## Case studies

In this section, we present examples to illustrate our three main areas of contribution: modelling and validation, simulation, and verification. Section 8.1 describes the use of p-models for several examples. In Sect. 8.2, we show how we can leverage p-models to improve automation in the development of simulations. Finally, Sect. 8.3 demonstrates the use of the semantics of p-models in conjunction with that of d-models to support the verification of properties of interest.

### Modelling and validation

**Fig. 23:**
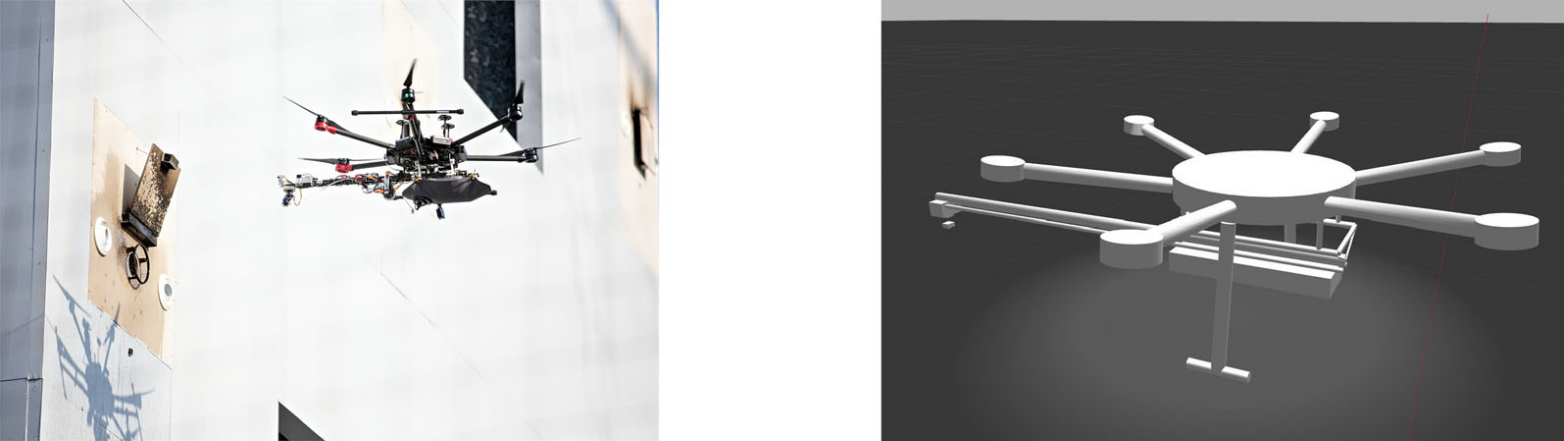
Firefighter: robotic platform and Gazebo rendering of the generated SDF document

Regarding the use of RoboSim for modelling and evaluation, we are concerned with demonstrating expressivity to capture common forms of robotic platforms. A full usability study with practitioners, however, is out of scope. On the other hand, RoboTool has been used to support the modelling of a variety of examples, including two large models described briefly here.

The implementation of the metamodel ensures that the p-model and platform mapping notations are well-formed; in that it is described according to the widely used *Eclipse Modeling Framework* (EMF) metamodel. Furthermore, as already mentioned, the validation rules implemented in our prototype are in one-to-one correspondence with the well-formedness conditions described here and in [51]. So, tests of RoboTool are evidence of the suitability of these conditions. We have a base of over 120 examples^20^ that cover all the constructs of the notation, and six larger case studies, some developed with external collaborators, two of which are presented here.

Each of the 120 examples focuses on a particular construct of the p-model notation. We arrive at 120 p-models to cover combinations of a construct with various cases of others involved in its definition. For instance, when considering a link, we have models for the various kinds of blocks to which they may be connected. The 120 p-models exercise all constructs of the notation, but cannot always be simulated in a meaningful way. The larger case studies, on the other hand, are for real robots. Three of them have not been written by a developer of the notation. Moreover, they cover a drone, two different robotic arms, and three different ground robots.

Below, we present two of the larger case studies: the robotic platforms for a firefighter UAV and a robot arm used for robot-assisted dressing.

***Firefighter UAV***

This case study is an unmanned aerial vehicle, shown on the left in Fig. 23, designed to fight fires.^21^ This example is inspired by the Challenge 3 from the MBZIRC 2020 competition,^22^ where multiple robots cooperate to extinguish fires in a building. Our robot approaches the building using its known GPS location, and upon fire detection, directs water to extinguish it. We have defined a RoboChart model that specifies the control software in terms of the services provided by an abstract robotic platform, and a RoboSim p-model that describes the details of this robotic platform and how its services are realised in terms of sensors, actuators and joints.

**Fig. 24:**
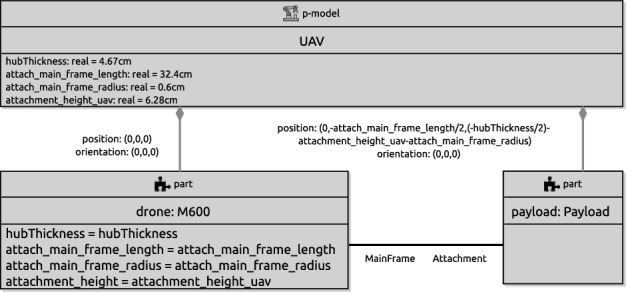
p-model of the Firefighter UAV

**Fig. 25:**
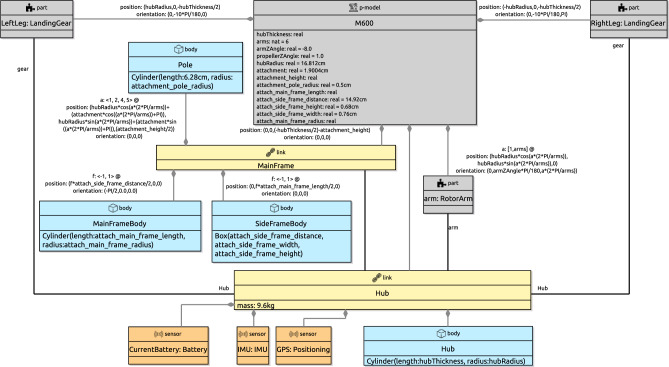
p-model of the drone component of the Firefighter UAV

Figure 24 shows the p-model that specifies the UAV; it declares a number of constants (for example, hubThickness, of type real and value 4.67cm) and it has two parts: drone, which is an instance of the p-model M600, and payload, an instance of the p-model Payload. These parts are positioned in such a way that the drone is at the centre, and the payload is attached to the bottom of the drone.

Figure 25 presents the p-model for the drone part in terms of its frame, three other parts (LeftLeg, RightLeg, and RotorArm), and a link Hub that houses three sensors: a battery monitor CurrentBattery, an inertial measurement unit IMU, and a global positioning system GPS.

**Fig. 26:**
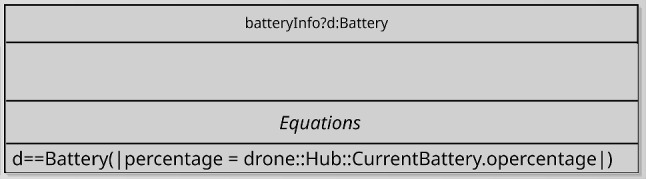
Event specification in the platform mapping of the Firefighter UAV

Figure 26 illustrates the definition of the event batteryInfo of the RoboChart model in terms of the sensor CurrentBattery of the p-model. In this definition, the value communicated through the event batteryInfo is defined as a record Battery, where the percentage attribute is set to the output opercentage of the sensor CurrentBattery.

The Gazebo rendering of this p-model, based on the SDF document generated, is on the right in Fig. 23. The complete model is available.^23^

The two top-level parts of UAV reflect a separation of concerns between the M600 and the custom Payload developed by our collaborators. The M600 p-model, whose definition is informed by a CAD design, is parametric, allowing, for example, instantiations with different numbers of rotor arms. The Payload p-model uses components of the RoboSim library to model servos and the depth and RGB cameras, whose calibration matrices have been obtained for the specific hardware used. The platform mapping exploits rich data types and nondeterminism to capture assumptions of the application at a high level of abstraction, for example, the maximum speeds of the drone.

**Discussion**. Overall, the UAV p-model consists of 43 blocks, of which 5 are indexed, yielding an equivalent total of 62 blocks. Indexation alone has saved us from defining another 19 (very similar) blocks, while the automatic generation of SDF in seconds has enabled an iterative approach to modelling and validation. While we have found that a good understanding of relative frame orientations is key to correctly position blocks in space, the results obtained by simulating the SDF in Gazebo have allowed rapid validation and iteration. In a matter of the couple of minutes that it takes to load the SDF document in Gazebo, carry out a visual inspection of the graphical display, fix the p-model, and regenerate the SDF, we can improve the model. In this way, we have, for example, found that in an earlier version of the model, two rotor arms were erroneously positioned at the same coordinates. Additionally, issues related to the positioning of links in the gimbal and inaccurate assignment of masses were identified during modelling.

Overall, RoboSim abstractions have been useful to deal with a rather large model in a concise and structured way. The facilities of the notation have also allowed us to record simulation assumptions often left implicit. On the other hand, in spite of the simplified and uniform approach to defining frames of reference, there is still significant effort involved in getting their coordinates right. The availability of support for rapid rendering is very useful, but it may be the case that actual animation of the platform is needed to reveal some modelling mistakes.

***Robot-assisted dressing***

This case study is a robotic arm that assists a potentially physically impaired user to put on a coat. The need for daily assistance with dressing is a reality for over 80% of nursing home occupants. Since this puts pressure on the limited capacity of care systems, the development of robotic solutions has been explored. Our case study is based on the system from [6] and the safety assessment results from [21]. The robotic platform is a Franka-Emika robotic arm^24^ with a custom gripper device for grasping a garment while dressing a user.

**Fig. 27:**
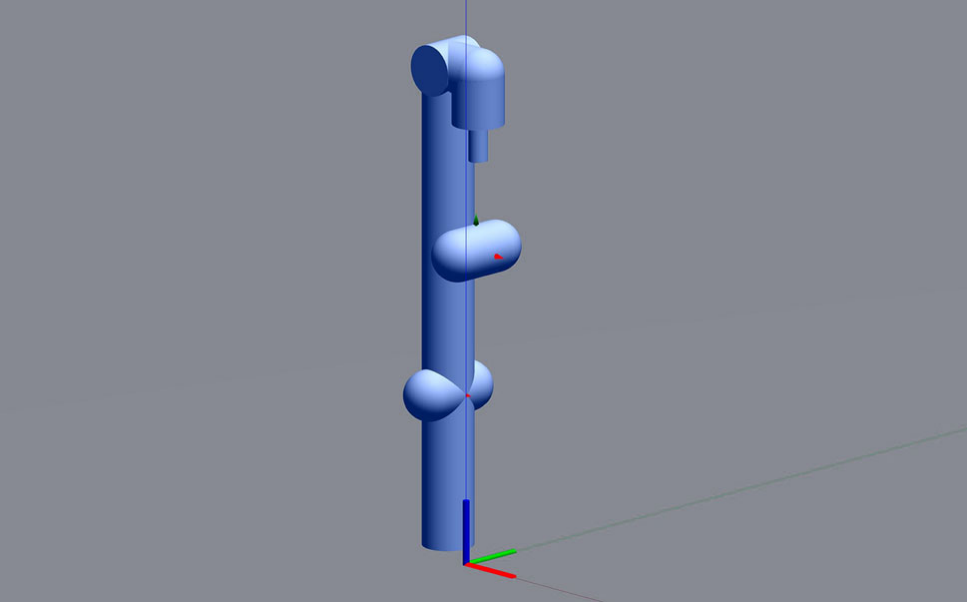
Rendering of the p-model of the robot-assisted dressing arm

We have defined a RoboChart model that specifies the control software and a RoboSim p-model that models the robotic arm and how the services used by the software relate to the sensors, actuators and joints of the robot. The d-model consists of one controller with six top-level state machines capturing different submodules of the system. The p-model (omitted here due to its size) captures the nine degrees-of-freedom Franka-Emika arm as nine links connected by eight revolute joints. Additionally, each link has a force sensor and each joint is actuated using a motor. The custom gripper is placed at the end of the arm alongside a six-axis force sensor. Figure  27 presents the Gazebo rendering of the p-model for the generated SDF. The complete p-model is available.^25^

**Discussion**. Overall, the p-model consists of 50 blocks. Despite the complexity of the model, RoboTool was able to generate the SDF model in under a second, allowing for rapid validation of the model in Gazebo. We found the overall structure of the RoboSim p-model to be a clear representation of the kinematic chain making up the arm, elucidating the underlying kinematics and the roles of each sensor and actuator. We have been able to utilise standard actuator and sensor types provided by the RoboSim library to generate the equations of the arm based on the specific parameters provided by the manufacturer. This high level of abstraction allowed us to rapidly tailor different variants of the model to match robot arms produced by different vendors in order to reproduce different robot-assisted dressing systems. The main limitation has been the need to specify absolute positions for each link of the arm, while relative joint coordinates may be an appealing representation of robotic arm poses.

***Final considerations***

Our notation imposes some restrictions, such as the format for the specification of orientations. On the other hand, it is general and flexible in other aspects, such as the specification of behaviour, and the allowed patterns of connections between elements, which includes closed kinematic chains. The techniques chosen to exploit our models may, however, impose restrictions. For instance, if translating p-models to URDF, closed kinematic chains cannot be directly supported. When translating p-models to SDF, on the other hand, we do not have this limitation, but must ignore the specified behaviours of joints, sensors and actuators and are forced to rely on whichever behaviours are encoded in the simulation platform.

Our case studies cover ground and air robots, and articulated arms. Developing p-models of underwater robots might shed light on other features of p-models. Moreover, more complex examples can push the limits of both RoboSim and RoboTool. Future work will also investigate translation from CAD models, which focus on mechanical and material properties, to p-models.

### Simulation

Regarding our contribution to simulation development, we are concerned with our set of rules, which define the RoboSim to SDF translation technique. We have sought evidence that there are enough rules to translate any RoboSim diagram, that the rules themselves are well-formed, and that the SDF documents they generate follow the SDF format definition. We are also concerned with the scalability of SDF document generation based on the rules. The scalability of the simulations depends on the simulator and physics engine, and it is not considered here, but we have had no problems in this regard in our extensive tests.

The adequacy of the rules has been established firstly by implementing them, thus demonstrating that they are both amenable to implementation and type correct, and secondly by a battery of tests. These include all 120 examples mentioned in Sect. 8.1 and the six larger case studies. They have all been translated into SDF using our tool, and the corresponding SDF models can be found in the associated pages^26^^,^.^27^ For each example, we have uploaded the resulting SDF document in CoppeliaSim, and inspected the result. This provides evidence that the resulting SDF documents are well-formed, and the rules capture the physical properties of the p-model elements appropriately.

Next, we discuss another case study, where the automatically generated SDF document has been used to simulate and test an industrial application.

***Manufacturing robot***

This case study is a robotic arm that uses a camera to identify parts in a conveyor belt, and pick and place them to manufacture devices of a given specification. We have developed a RoboChart model of the control software, a cyclic refinement of the RoboChart model defined by a d-model, and a p-model that describes the arm in terms of its links, joints, sensors, and actuators.

**Fig. 28:**
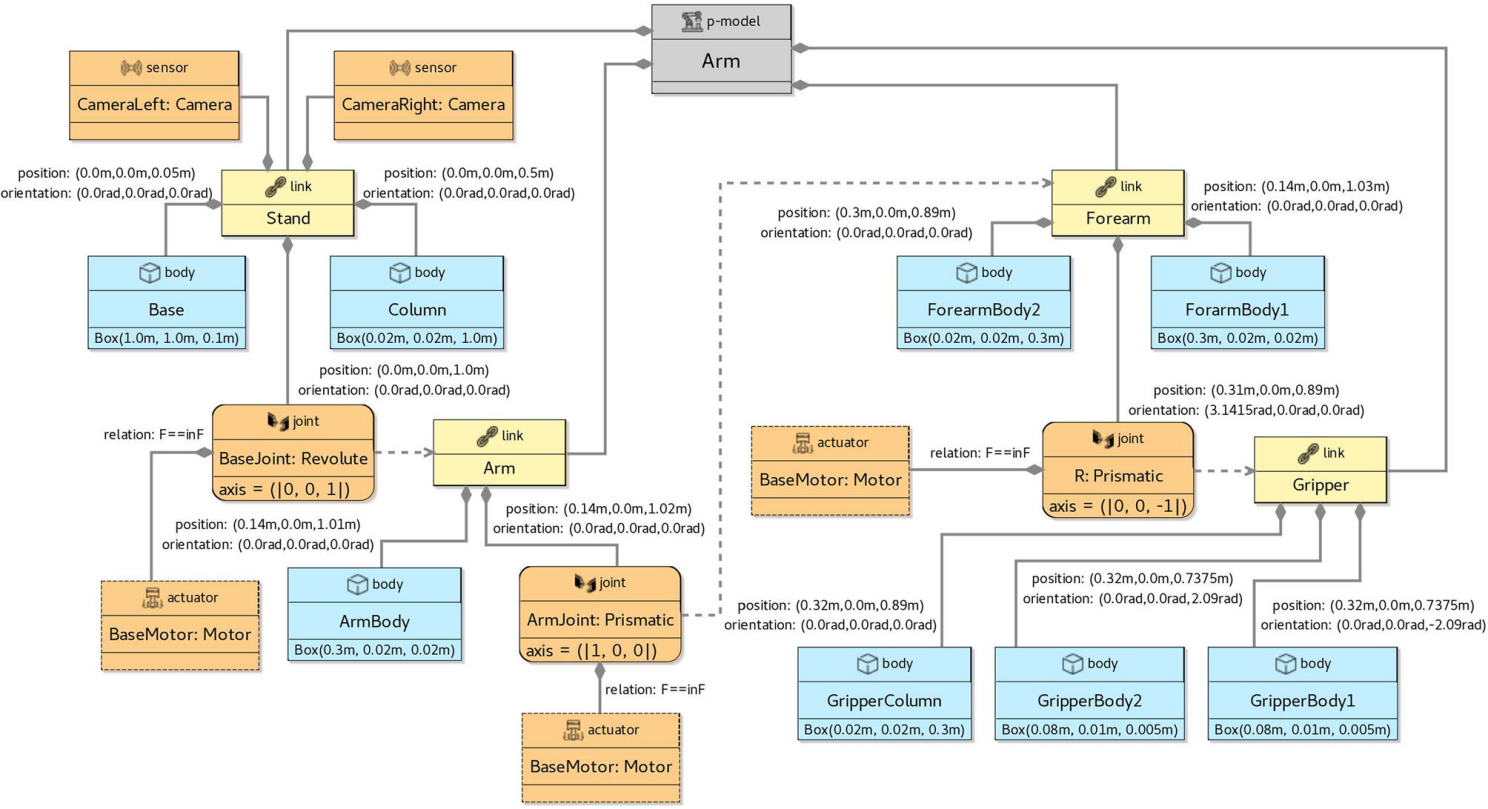
P-model of a robotic arm used in the manufacturing robot case study

Figure 28 shows the p-model Arm containing four links, Stand, Arm, Forearm, and Gripper, with the first two connected through an actuated (BaseMotor) revolute joint (BaseJoint), and the last three links connected by two actuated prismatic joints. Each of the links includes information about their geometric realisation via bodies. Overall, the p-model consists of 22 blocks, covering the main elements of a p-model.

**Discussion**. In general, establishing the position and orientation of links can be challenging without visual feedback, but in this particular case study, this task was straightforward due to the number of components and the limited number of variations in orientation. Additionally, we have reused the joints from the RoboSim library and specified tailored models of sensors and actuators.

The p-model has been used in conjunction with the d-model to automatically generate the SDF document and a simulation of the control software. The result has been successfully used as a basis to develop and evaluate a testing approach that relies on a variety of models, including RoboChart and RoboSim models, to generate test scenarios and evaluate them using simulation. In particular, the SDF^28^ and software generated have been used to run the test scenarios in CoppeliaSim.

***Final considerations***

CoppeliaSim has a model editor that provides 3D rendering and so immediate visual feedback. For p-models, we have a graphical editor and need to generate SDF to obtain visual feedback. In a 3D editor, it is easy to position elements of the model, but it requires effort to define those positions precisely. Being able to input and update the model in a simple and direct way more than compensated for the extra effort of generating the SDF and loading it into CoppeliaSim for visualisation.

Table 4 summarises information about the size of the SDF documents generated and translation time for our cases studies in the previous section and for the manufacturing robot.

**Table 4:** Data on SDF documents: generated using version 1.0.0.202409270944 of the p-model SDF generator in Eclipse 2021-12 running on Ubuntu 22.04

**Case study**	**p-model blocks**	**SDF document lines**	**Translation time**
FirefighterUAV	39	1952	57 ms
marXbot	49	2471	73 ms
Robot-assisted dressing	54	838	41 ms
Assembly Line	21	428	40 ms

### Verification

Here, we are concerned with our set of rules to generate *CyPhyCircus* models. We seek evidence that there are enough rules, that they are well-formed, and define well-formed *CyPhyCircus* programs. We are also concerned with the scalability of the generation of *CyPhyCircus* programs and their use in verification. Further work on automation of verification techniques for *CyPhyCircus*, however, is needed. At the moment, we use a mechanisation of *CyPhyCircus* in Isabelle/UTP.

The implementation of the semantic rules from Sect. 6 demonstrates that they are amenable to implementation and type correct. In addition, the use of the *CyPhyCircus* parser determines the validity of the mathematical models defined by the rules for our examples. Finally, a further case study described here uses the p-model of a mobile robot and the encoding of its semantics to establish a property of the robotic system. This case study provides some evidence that the semantics of RoboSim p-models can be used in the verification of properties, but, as noted, further work on proof automation is still needed.

**Fig. 29:**
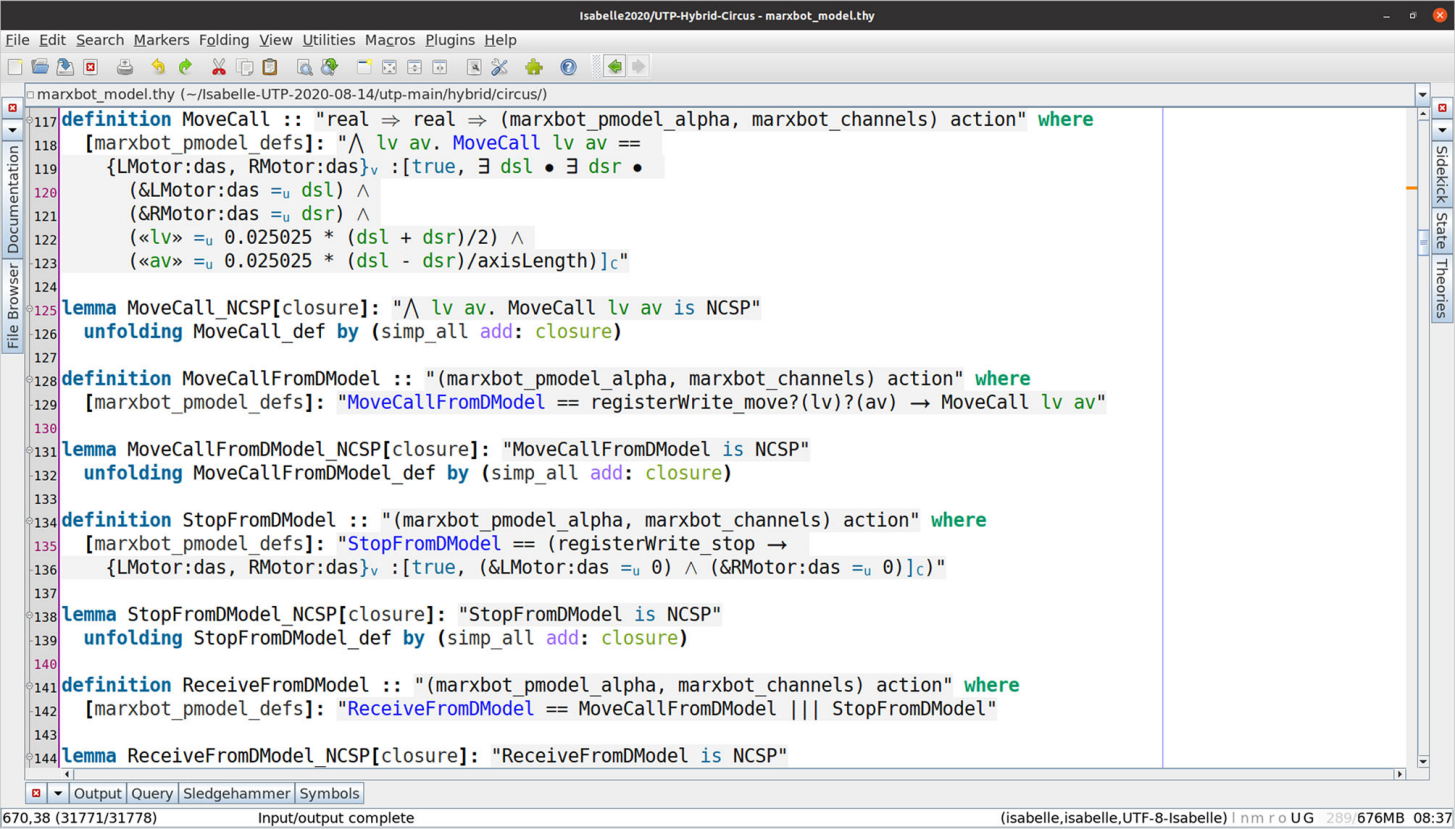
Isabelle/UTP rendering of part of the *CyPhyCircus* process in Fig. 18

***marXbot***

The generated $\textsf {CyPhyCircus}$ models are appropriate for use with the theorem prover Isabelle/UTP [31]. Figure 29 shows the Isabelle/UTP encoding of the actions *MoveCall*, *MoveCallFromDModel*, and *ReceiveFromDModel* also included in Fig. 18. Such encodings can be generated by a simple text-to-text transformation: we note how close the Isabelle/UTP notation is to that used in pen-and-paper *CyPhyCircus*.

A few differences are of note. First, Isabelle requires that definitions be introduced before use. This is not required by *CyPhyCircus* (as usual in Z-based notations), and a top-down presentation is often adopted like in Fig. 18. It is, however, required that a *CyPhyCircus* specification can be rewritten following a define-before-use order. So, the order of the *CyPhyCircus* definitions in the semantics of a p-model does not impose any challenge for producing an Isabelle/UTP encoding.

Second, each definition of the encoding corresponds to a term of a Isabelle/UTP theory that must satisfy some healthiness conditions. These need to be provided as lemmas, which in Fig. 29 are labelled [closure]. Their proof is a fairly direct application of healthiness-condition closure laws, and can be automated. (The lemmas all have the same form, and by unfolding the definition and applying the closure laws, we get a proof).

To prove a property in Isabelle/UTP, we need to describe it in terms of a precondition, a postcondition, and a pericondition. A precondition restricts the initial state of the system. For our example, we have the following precondition.

$$Proximity0\_distance(time) > sensorThreshold$$ and lvel $$< maxSpeed$$

Here, we consider constants *sensorThreshold* and $maxSpeed$ that record the minimum distance of an obstacle before it can be sensed by the robot, and the maximum speed of the robot. The precondition requires that the robot does not start near an obstacle, and never reaches a speed above the limit *maxSpeed*. In the initial state, the robot is stationary, but with this precondition, our result applies from any state in which the robot may be already moving, as long as it is not right up against the obstacle already, and does not move too fast. We recall that lvel is a d-model constant recording the linear speed of the robot. The state variables, as mentioned, are functions of time, and we use $Proximity0\_distance(time)$ to refer to the value of $Proximity0\_distance$ at a particular instant *time*.

A postcondition restricts the behaviour of the robotic system when it terminates. Typically, and in our example, it does not terminate, and so the postcondition is just *false*. More interesting is the pericondition, which restricts the behaviour once the precondition is satisfied. For our example, the pericondition is below, where we refer to the p-model process variables $Proximity0\_distance$, $LMotor\_T$, $RMotor\_t$, and an extra variable *t* of the Isabelle theory to represent the current time.

For every sample time *t*0 (multiple of *cycle*) such that

$$\quad Proximity0\_distance(t0) \le sensorThreshold$$

there is a time *t*1 such that

$$\quad LMotor\_T(t1) =_{\epsilon } 0.0$$,

$$\quad RMotor\_T(t1) =_{\epsilon } 0.0$$, and

$$\quad t0 \le t1 < t0 + t$$

Here, we note that equality $=_{\epsilon }$ is parametrised by a tolerance $\epsilon$. In our proof, $\epsilon$ is $10^{-9}$. To automate the proof, we ally the use of Isabelle/UTP^29^ with a model checker Flow* [23].^30^ The encoding of our model in Isabelle/UTP, as well as proof artefacts that explain how Isabelle/UTP and Flow* are used in conjunction, are available.^31^

## Conclusions and future work

We have presented RoboSim p-models, a novel graphical notation for the description of physical artefacts: parts and robotic platforms, and their connection to a control software model via a platform mapping. Unlike XML-based notations, RoboSim block diagrams embed concepts that facilitate the design of readable and reusable models. A large set of well-formedness rules characterise valid models and provide guidance for modellers. Still, using RoboTool, we can automatically obtain SDF documents and process-algebraic mathematical descriptions of a p-model and associated platform mapping for use with simulators and verification tools. Adaptation of the work to deal with a variety of simulators and other XML-based notations is straightforward, given our precise description of the translation technique via transformation rules. In particular, adapting our approach to generate URDF is primarily a technological issue, with a significant proportion of the existing model-to-text transformation potentially reused.

The definition of a behaviour for the joints, sensors, and actuators in a RoboSim block diagram provides a natural point to link RoboSim p-models to models in other notations. For sophisticated actuators or sensors, instead of explicit equations, we may want to use control law tools and simulators. For example, Simulink is a widely used tool for which verification facilities compatible with those of RoboSim are available [14]. To have the behaviour of a RoboSim block defined by a Simulink diagram, instead of equations, is an attractive option. Integration of RoboSim d-models and Simulink is explored in [54].

While, in general, SDF could be used directly to create physical models, RoboSim p-models allow the explicit specification of behaviours, which is not currently supported by SDF, and is desirable for documentation and required for verification. When we translate a p-model to SDF and use a simulator, the physics engine adopted defines the simulated behaviour. As such, the use of simulations in our approach relies on existing simulators, and therefore, we do not directly address the details of how a simulation is run internally; this is the role of the physics engine. Compatibility with the p-model equations is not guaranteed, since different engines embed different assumptions. Moreover, for optimisation, the behaviour specified in RoboSim may not be adequate for simulation. With a p-model, however, we have documentation of the expected behaviour, which is in itself a tool to understand the faithfulness and threats to the validity of the simulation. Further work is exploring the generation of simulations using a physics engine that gives transparency in terms of the equations that it uses.

Compatibility between simulation and mathematical models can also be affected by annotations. In Fig. 5, for instance, the value of the xyz attribute of axis can be entirely different from the value of the constant AXIS in LHinge. In this case, the definition of LHinge and the model obtained by translation to SDF do not match. This feature can be useful to explore various simulations, and we, therefore, do not enforce compatibility.

Once the values of all constants of a p-model are defined, it is possible to enforce additional well-formedness conditions. For example, we can make sure that the value for the transparency of a BodyDefinition is a real value between 0 and 1. Such checks, along with possible checks of compatibility of values of attributes in the p-model and in the annotations, are left as future work. As opposed to the well-formedness conditions presented here and enforced in RoboTool, these extra checks are dependent on the semantics of SDF.

As said, RoboChart and RoboSim d-models are related: a refinement relation allows the comparison of RoboChart and RoboSim d-models, and an automatic refinement strategy from RoboChart models to RoboSim models is under development [16]. Additionally, a new notation, RoboWorld [4] has been developed to support the abstract specification of operational requirements and interaction patterns between robots and the environment. Within the RoboStar framework, RoboWorld sits at the same level as RoboChart models. As such, a natural direction for future work is to establish the relationship between RoboSim p-models and RoboWorld.

The main line for future work, however, is the additional exploration of verification tools to use *CyPhyCircus* models to prove properties of the robots that depend on their physical bodies. With this, besides generation of low-cost simulations, RoboSim will enable verification with a higher level of assurance. Our results show promise in the use of Isabelle/UTP, a well-established theorem prover, in conjunction with a model checker to improve automation. We will investigate how model checking and theorem proving can be combined to not only optimise the verification, but also to improve understanding of why proofs fail and how models can be changed to facilitate verification. Finally, we will explore additional proof strategies using a variety of model checkers.

In current work, we are integrating RoboSim and its sister languages with existing commercial tools for testing and proof, including using p-models to support testing with hardware-in-the-loop. We are also pursuing industrial demonstrators in an effort to encourage acceptance in industry of our approach and, perhaps more importantly, model-based approaches to software engineering.

Overall, in future work, it will be important to compare our approach to those enabled by other simulators widely used by roboticists. Full evaluation experiments will be needed to provide data to evidence the improvements brought about by RoboSim and its associated techniques.

## Footnotes

1 http://sdformat.org

2 www.ros.org

3 www.mathworks.com/products/simulink

4 http://pybullet.org

5 http://dartsim.github.io

6 https://www.khronos.org/collada/

7 www.cs.york.ac.uk/robostar/tools/

8 www.cs.york.ac.uk/robostar/case_studies/

9 www.eclipse.org/capella/

10 http://wiki.ros.org/xacro

11 www.cs.york.ac.uk/robostar/case_studies/

12 http://robostar.cs.york.ac.uk/case_studies/marxbot/

13 http://robostar.cs.york.ac.uk/case_studies/marxbot/

14 www.cs.york.ac.uk/circus/RoboCalc/robotool/

15 www.eclipse.org

16 www.eclipse.org/xtext

17 www.eclipse.org/sirius

18 A dialect of Java (www.eclipse.org/xtend/)

19 http://lua.org

20 https://github.com/UoY-RoboStar/pmodel-examples

21 https://robostar.cs.york.ac.uk/case_studies/firefighting-UAV/index.html

22 https://www.youtube.com/watch?v=S0Ok2cnselU

23 https://github.com/hlsa/Verifiability-Node/tree/main/Models/FirefightingDrone/RoboSIMModel

24 https://franka.de/

25 https://github.com/hlsa/Verifiability-Node/tree/main/Models/RAD

26 https://robostar.cs.york.ac.uk/case_studies/firefighting-UAV/

27 https://robostar.cs.york.ac.uk/case_studies/assisted-dressing/

28 https://robostar.cs.york.ac.uk/case_studies/assembly-line/

29 Isabelle/UTP version provided by commit c9122f143509aebc2ca273990a1e7c1a70609628 of repository https://github.com/isabelle-utp/utp-main.

30 Flow* version 2.0.0.

31 www.cs.york.ac.uk/robostar/case_studies
